# Network Indices and Diversity Follow Distinct Patterns for Bee–Plant Networks Along a Neotropical Elevational Gradient

**DOI:** 10.1002/ece3.74549

**Published:** 2026-10-27

**Authors:** Valerie E. Peters, Jenny Hsu

**Affiliations:** ^1^ Department of Biological Sciences Eastern Kentucky University Richmond Kentucky USA

**Keywords:** Anthophila, biodiversity, ecological networks, environmental gradient, pollination

## Abstract

A critical goal of ecology is to quantify mutualistic network structure across all ecosystems and organisms, as these networks often represent essential ecosystem services and therefore network metrics are often used as conservation targets. Some major gaps still exist, one of which is Neotropical bees, a group expected to contribute exceptional insight to ecological network theory, owing to the extreme diversity of mutualistic interactions characteristic of the Neotropics, as well as the unique global distribution patterns of bees. Elevational gradients are a useful tool for quantifying change in network structure, with results having direct implications for predicting resilience in ecosystem service provisioning. In this study, we compared mutualistic network properties of 28 Neotropical bee–plant networks, along an elevational gradient from 200 to 1500 m elevation. Bee species richness peaked at mid‐elevation while network specialization and modularity decreased with increasing elevation. Connectance and nestedness showed a statistically significant increase by elevation. Combining indicator analysis and the number of bee species collected from only one elevational band, the majority of bee species, 101 of 157 (64%), showed association with specific altitudes, and each elevational band harbored a unique assemblage of bee species, with 95% of beta‐diversity explained by species replacement. The highest elevation band studied represents the top elevation of these short tropical mountains, emphasizing an urgent conservation need for these bee communities throughout the tropical realms as temperatures rise. Our finding of high specialization in lowland mutualistic networks of the tropics suggests that previously documented patterns of higher generalism toward lower latitudes may not have included bee–plant networks from Neotropical lowland and mainland ecosystems; resolving this debate will have important implications for conservation and restoration action.

## Graphical abstract

Ecological studies of how interactions, communities, and species change along elevational gradients are still missing from some key organisms and ecosystems. We quantify bee–plant interactions and bee diversity patterns along a low elevation Neotropical mountain. Bee–plant networks were characterized by very specialized and modular at low elevations, decreasing linearly with elevation while bee richness peaks at mid‐elevation, and high species replacement at each elevational band.

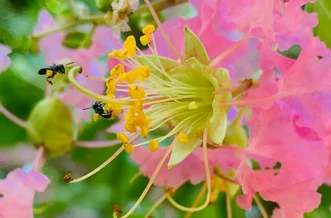

## 1 Introduction

Ecological interaction networks illustrate specific ecosystem functions, for example, pollination, seed dispersal, or predation (Kaiser‐Bunbury and Blüthgen 2015). An emerging challenge in ecology of vital importance for human well‐being is understanding the relationship between structural patterns of these interaction networks and ecosystem function and stability (Tylianakis et al. 2010; Benadi et al. 2013; Andresen et al. 2018; Lázaro et al. 2020; Acevedo‐Quintero et al. 2020; Vázquez et al. 2022). The integration of ecological network theory (Ings et al. 2009; Landi et al. 2018) with biodiversity conservation and its applications such as ecological restoration and agroecology cannot be effective until we resolve these relationships; including which network structural elements or indices best represent ecosystem function and stability, whether positive or negative values of these indices are conservation targets, and whether relationships vary by taxa and along environmental gradients (Heleno et al. 2012; Harvey et al. 2017; Vizentin‐Bugoni et al. 2018; Pinheiro et al. 2023; Wang et al. 2024). Current literature supports mixed results regarding the direction of the relationship between the network indices of specialization, connectance, modularity and nestedness and network stability and function, with some studies finding that increasing values of these indices confer greater network stability and function while others have found lower values to be associated with higher network stability and function (Thébault and Fontaine 2010; Heleno et al. 2012; Benadi et al. 2013; Hoiss et al. 2015; Bascompte and Scheffer 2023; Domínguez‐Garcia et al. 2024). Ecological network science cannot resolve these debates without filling the gaps in knowledge caused by underrepresented network types, for example, an uneven global distribution of studies biased toward non‐tropical areas (Vizentin‐Bugoni et al. 2018). Invertebrate mutualists of lowland, low‐ and mid‐latitude ecosystems have been especially underrepresented in ecological interaction network studies (Vizentin‐Bugoni et al. 2018; Wang et al. 2024), and the tropics have been hypothesized to host both more specialist (Pellissier et al. 2018; Classen et al. 2020) and more generalist (Schleuning et al. 2012) mutualistic interactions. Of all tropical realms, the Neotropics is home to an unrivaled diversity of angiosperms and their mutualistic animal partners (Fleming and Kress 2013, Roubik 2024), with invertebrate mutualists notably understudied (Freitas et al. 2009).

Pollinating insects are one of the most important groups of invertebrate mutualists to humanity for their role in the maintenance of plant diversity (Balvanera et al. 2005; Ollerton et al. 2011) and food security (Potts et al. 2016). In the Neotropics, animals pollinate approximately 98%–99% of all flowering plant species (Bawa 1990) and 70% of all crop species, with bees (Hymenoptera: Apoideae: Anthophila) being one of the most important taxa of pollinators (Roubik 1995). Globally, native bees pollinate crops more effectively than domesticated honeybees (Goulson et al. 2015). In recent years, native bees have been declining all over the world (Biesmeijer et al. 2006; Freitas et al. 2009; Martins et al. 2013; Theisen‐Jones and Bienefeld 2016; Robinson et al. 2017; Prendergast et al. 2023). Bee declines, particularly in rare species, can affect the pollination services of wild plants and crops with ecological and economic consequences (Potts et al. 2010; Zattara and Aizen 2021). Causes for bee decline include invasive species, diseases, climate change, habitat loss, and the use of pesticides (Goulson et al. 2015; Robinson et al. 2017). While critical conservation research continues to increase knowledge of which factors contribute most to the observed declines in native bee populations (e.g., Ulyshen et al. 2023), there is also an urgent need to identify patterns in bee communities and bee–plant networks along environmental gradients, so that conservation practitioners can develop effective initiatives that protect both wild bee communities and their pollination services (Bascompte and Scheffer 2023; Figure 1). Only recently have bee species richness patterns been documented latitudinally (Orr et al. 2021) while altitudinal patterns of bee communities and bee–plant interaction networks from low‐ and mid‐elevation tropical ecosystems remain undescribed despite that tropical countries are expected to be among the most vulnerable to pollination service loss owing to their higher dependence on food provisioning and poverty level (Ashworth et al. 2009).

**FIGURE 1:**
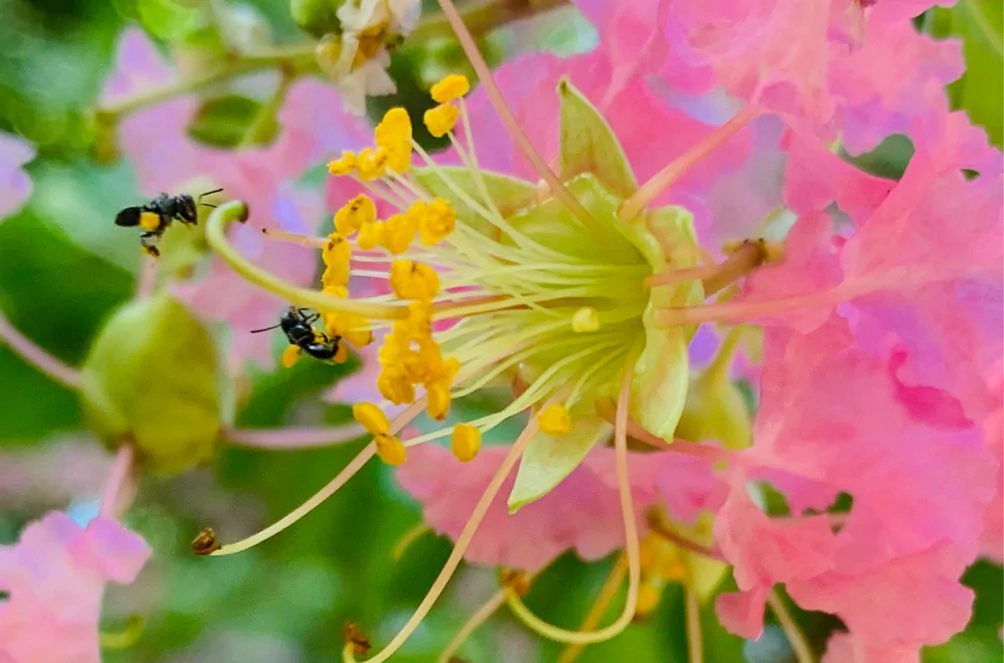
Organism photograph showing smallest bee species found in study ( *Trigonisca pipioli* or *Dolichotrigona schulthessi* ) foraging on pollen of *Lagerstroemia* species. Both species were only collected from L, LM, and M, or L and LM elevational bands, respectively.

Elevational is one type of environmental gradient that provides variation in abiotic factors over relatively small spatial scales while reducing the influence of potentially confounding effects such as the variation in biogeography, history, and day length compared to latitudinal and other environmental gradients (Hodkinson 2005). Elevational gradient studies are also essential to understanding spatial patterns and drivers of ecological interaction network architecture, as well as for predicting the vulnerability of communities and ecosystem functions at different elevations (Hoiss et al. 2015; Chesshire et al. 2021). Elevational gradients can also serve as models to predict phenological changes in the case of interaction mismatch (Hoiss et al. 2015), providing insight into the diet breadth of pollinators, a topic that remains relatively unknown (Lara‐Romero et al. 2019). Tropical elevational gradient studies of pollination networks are scarce and tend to focus on higher elevations (Watts et al. 2016, Classen et al. 2020, Pelayo et al. 2021, Dzekashu et al. 2023, Jacob Cristóbal‐Perez et al. 2024); partial networks that include mostly vertebrates like hummingbirds (Maglianesi et al. 2015; Vizentin‐Bugoni et al. 2018); and the Paleotropics (Adedoja et al. 2018; Albrecht et al. 2018; Classen et al. 2020; Dzekashu et al. 2023). Tropical high elevations differ abiotically from tropical lowlands, with conditions becoming colder and harsher, leading to an expected decrease in floral and faunal diversity (Classen et al. 2015; Jacob Cristóbal‐Perez et al. 2024). Indeed, lower bee visitation rates to flowers have been documented at high elevations (i.e., > 1800 m elevation) in the tropical Andes due to harsh abiotic conditions (Dellinger et al. 2021). These tropical elevational gradient studies of pollination networks have documented increasing, decreasing and no change in network indices with elevational increases, as well as seasonal differences in these relationships (Table S1). Tropical elevational gradient studies on bee richness patterns have found a continuous decline in overall species richness (Classen et al. 2015; Perillo et al. 2017), as well as abrupt declines in species richness for orchid bees, Tribe Euglossini (> 1200 m elevation) (Pinto et al. 2019); however, the lowest elevation included among all three studies was 870 m.

In this study we compare quantitative bee–plant interaction networks along an elevational gradient in the tropical Pacific slope of Northwestern Costa Rica. The lower and mid elevations of the gradient were classified as Tropical Dry Forest life zone (TDF). The TDF is a water‐limited biome, the most endangered tropical forest type (Stan and Sanchez‐Azofeifa 2019), and expected to harbor the most at‐risk species assemblages under modified climate scenarios owing to drastic alterations in precipitation regimes (Mccain and Colwell 2011). At mid to mid high elevations of the gradient, Tropical Premontane Forest life zone (TPF) replaced TDF, while the highest elevations were classified as Tropical Montane Forest (TMF). We assessed differences in bee species richness and the interaction network indices of connectance, modularity, nestedness and specialization along an elevational gradient from 200 to 1500 m a.s.l. on a mountain located in the Tilarán mountain range where the highest peaks lie between 1500 and 2000 m a.s.l., comparatively much lower to the Central and Southern Costa Rica mountain range peaks (Barrantes 2009). Within this elevational gradient, replicate bee–plant networks were constructed at five elevational bands to increase statistical power. Bee richness was expected to decline with elevation according to previous research (Guo et al. 2013; Classen et al. 2015; Perillo et al. 2017) due to environmental filtering of community members to only those able to tolerate the harsh conditions of high elevations (Dellinger et al. 2021). Based on our previous work in the southern lowlands of Costa Rica (Hinton and Peters 2021), we hypothesized that modularity and specialization would also decrease with elevation, while connectance and nestedness were expected to increase since they often are typically inversely related to specialization. To our knowledge, our study is the first to quantify bee species richness patterns and the bee–plant network indices of specialization, connectance, modularity and nestedness along the full extent of a low elevation Neotropical elevational gradient.

## 2 Methods

### 2.1 Study Area

The study was carried out along an elevational gradient located in the Tilarán Mountain Range in the Puntarenas province of Northwestern Costa Rica (10°15′57.96″ to 10°17′44.52″ N, −84°49′55.56″ to −84°48′14.4″ W). The elevational gradient is located on the Pacific slope of the Tilarán Mountain Range, and encompasses three life zones: TDF; 200–800 m a.s.l., TPF; 800–1200 m a.s.l., and TMF; > 1200 m a.s.l. Annual precipitation and moisture from cloud immersion varies dramatically among the three life zones, and complete turnover in tree and bird communities over short distances due to the strong moisture gradient have been documented (Haber 2000; Jankowski et al. 2009). Precipitation on the upper slopes can reach 7000–8000 mm annually and declines to 2000 mm toward the Pacific, with most precipitation falling from mid‐May to November and annual temperatures decreasing 6°C with every 1000 m altitude (Opler et al. 1980; Jankowski et al. 2009).

### 2.2 Monitoring of Plant–Pollinator Interactions

We established 28 plots of ca. 0.25 ha distributed within five elevational bands along an elevational gradient. Each plot comprised planted ornamental and crop species as well as naturally growing trees, shrubs and herbaceous plants typical of tropical countryside habitats. Landowner permission was obtained to collect all flower‐visiting bees observed on the plant species for six plots from within or near the following four elevational bands: low‐mid (LM), 500–600 m a.s.l.; mid (M), 800–900 m a.s.l.; mid‐high (MH), 1100–1200 m a.s.l.; and high (H), 1400–1500 m a.s.l., and four plots from the lowest elevational band: low (L), 200–300 m a.s.l. Each plot was separated from all other plots by at least 250 m to ensure spatial independence (Figure 2). Plots differed in plant species composition based on landowner decisions, but many plant species were common including fruit trees such as *Musa acuminata* (banana, plantain, cuadrado), *Psidium guyaba* (guava), *Byrsonima crassifolia* (nance), *Averrhoa carambola* (starfruit); crops such as corn, beans, *Sicyos edulis* (chayote), *Oscimum basilicum* (basil); ornamental plants such as *Trimezia steyermarkii*, *Stachytarpheta* spp., *Cosmos sulphureus* , *Zinnia peruviana* , *Salvia splendens* , *Begonia* spp., *Euphorbia* spp., *Hamelia patens* , *Jatropha* spp., *Pleroma* spp., *Streptosolen* spp., *Caesalpinia pulcherrima* , *Lantana* spp., *Impatiens* spp., *Turnera* spp., and herbaceous cover such as *Tripogandra serrulata* , *Asclepias curassavica* , *Melampodium perfoliatum* , and *Mimosa pudica* . Most studies assessing species interaction patterns look at ecological network structure in a specific place and at a specific time (Vázquez et al. 2022), and each network is typically delineated within a local, and feasibly sampled spatial scale, for example, 3, 30‐m transects (Aguirre and Junker 2024). Therefore, unless study plots are experimentally planted, this means that the plant community sampled will vary greatly between experimental units, especially in diverse tropical habitats. In this study, we used replication within each elevational band to control for the variation in plant communities between experimental units, if plant communities were structuring network structure more than elevation then standard errors around the means would be large and no statistical differences in network structure between elevational bands would be detected. An additional plot was sampled at the MH elevation that was used for species richness analyses but was excluded from network analyses due to the incomplete sampling at this location.

**FIGURE 2:**
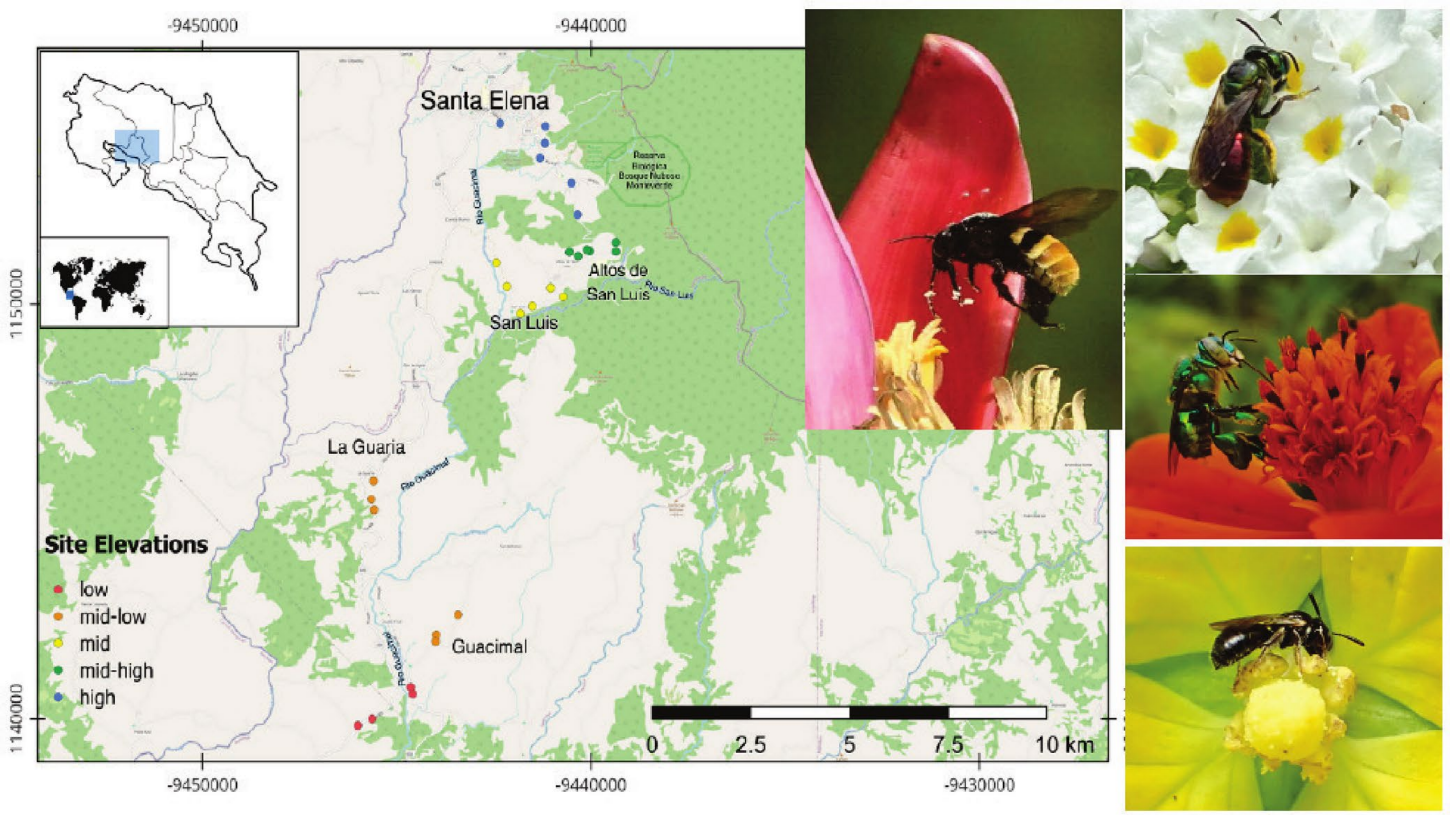
Map of 28 home garden plots in five elevational bands along a Neotropical elevational gradient in the Puntarenas Province, Costa Rica. Bee–plant interaction examples shown in photos adjacent to study area map. Photos from left to right, *Eulaema* species collecting pollen from *Musa* species (photo credit: Paula Foley); *Ceratina eximia* visiting *Lantana velutina* ; *Euglossa* species visiting *Cosmos sulphereus* (photo credit: Evie Cecil); *Ceratina* species visiting *Ludwigia* species (photo credit: Stan Rullman).

The censuses were carried out from 27 May to 21 July 2023, between 08:00 and 12:00 h, to avoid periods of heavy rain which typically occur in the afternoon, however the rainy season was very delayed in 2023 and did not begin until 31 May with infrequent rain subsequently. All plant species with open flowers in the plots were censused for at least 15 min during which all bees landing on the reproductive parts of the flower were netted for collection. If no bees were collected from the flowers, the plant was not included in the network. Each plot was censused four times in 2023 by four to five observers with duration of sampling lasting 1–1.5 h depending on the number of flowering plant species. Multiplying the number of observers with the sampling duration across each of the four sampling periods per plot, a total of approximately 560 observation hours were spent during 35 observation days. All collected bees were preserved in ethanol and returned to Eastern Kentucky University for processing and identification. Stingless bees (Tribe Meliponini) were identified to species following (Jarau and Barth 2008) and unpublished keys from R. Ayala and *M. Fernandez* . Bees of the Tribe Tapinotaspidini were identified to species using (Aguiar 2009, 2018; Aguiar and Melo 2011). Male orchid bees (Tribe Euglossini) were identified to species using (Roubik and Hanson 2004). Large carpenter bees (Tribe Xylocopini) were identified to species using Mawdsley (2017). Halictidae, Eucerini, and female orchid bees were identified to species or morphospecies using the reference collection at the University of Kansas Snow Museum of Natural History and (C. D. Michener 1954; Engel 2000, 2014; Ayala Barajas and Griswold 2012; Gardner and Gibbs 2020; Melo and de Liz 2025). All other bees were identified using a reference collection in the Peters lab and (C. D. Michener 2010; Hanson et al. 2023). All specimens are housed in the Peters lab at Eastern Kentucky University. Plants were identified to species or morphospecies in the field using iNaturalist, local expert help, and field guides (Gargiullo et al. 2008; Zuchowski 2022).

### 2.3 Network Indices

Quantitative data of bee species visitation frequencies on different plant species were used to estimate all network indices, as these are considered more precise compared to presence–absence interaction data (Dormann and Strauss 2014). All network indices were calculated using the R package “bipartite” (Bluethgen et al. 2017). The following indices were quantified along the elevational gradient (see Table S2 for network index definitions):

Specialization (H2′): Specialization was calculated as the degree of network specialization (H2′) which characterizes the average degree of specialization of the species in the network and ranges from 0 to 1, with higher values indicating higher network specialization (Blüthgen et al. 2006). Specialization was calculated for each plot.

Weighted Connectance: Connectance was calculated using the weighted connectance algorithm, as this algorithm is most appropriate for quantitative data. Weighted connectance values range from 0 to 1 and describe the level of generalization in a network by quantifying the number of realized interactions in a network divided by all possible interactions (Bersier et al. 2002). Weighted connectance is also a way to quantify interaction diversity in a network.

Modularity (Q): Modularity was calculated using the ModularityQ algorithm. Modularity measures compartmentalization in the network, quantifying the degree to which subsets of species interact more frequently with each other as compared to other species in the network (Dehling 2018). Modularity ranges from 0 to 1, where higher numbers represent more compartmentalization in the network.

Weighted Nestedness (NODF): Nestedness was calculated using the weighted nestedness overlap and decreasing fills, or weighted NODF, algorithm, which is most appropriate for quantitative interaction data (Almeida‐Neto and Ulrich 2011). Weighted NODF ranges from 0 to 100, with higher values indicating a decreasing structure of nestedness in the network, meaning that there is a greater likelihood of specialists interacting with generalists as well as among each other.

### 2.4 Data Analysis

All statistical analyses were performed in R version 4.3.2 (R Core Team 2025).

Estimates of true species richness in assemblages are especially challenging in species‐rich assemblages such as tropical life zones (Chazdon et al. 2022). Currently, the most statistically robust approach to address this challenge is to quantify sample coverage as a measure of sampling completeness, and then subsequently use the sample coverage to rarefy or standardize species richness estimates (Chao and Jost 2012). Sample coverage is defined as the fraction of the individuals in the assemblage, including undetected species, that belong to species represented in the sample, and is a quantitative way to measure sampling completeness and to standardize estimates of species richness (Chazdon et al. 2022). To quantify sampling completeness, sample coverage was quantified for each elevation band. To evaluate bee species richness patterns along the elevational gradient, we generated two types of rarefaction curves for each elevation band: those standardized by abundance and those standardized by sampling completeness or coverage (Chao and Jost 2012). A Chao1 abundance‐based species richness estimate was also generated for each plot, and an a priori planned contrast was used to test if estimated bee species richness differed by elevation using the R package “multcomp” (Hothorn et al. 2008). Chao1 and ACE abundance‐based estimates were also generated for each elevation band, pooling data from all plots per elevation band. Rarefaction curves, sample coverage or completeness, and abundance‐based species richness estimates were computed using the R packages “iNEXT” (Hsieh et al. 2025) and “vegan” (Oksanen et al. 2001).

Bee community composition similarity was quantified along the elevational gradient using bee abundance data. Significance of within and between plots similarity by elevation was calculated with the anosim function using the chao distance metric in the R package “vegan” (Oksanen et al. 2001). Bray–Curtis dissimilarity in the bee community between elevational bands was partitioned into nestedness and replacement components using the R package “betapart” (Baselga 2013). Statistical significance of similarity in bee species community composition between elevations was quantified using cluster analysis in the R package “pvclust” (Suzuki et al. 2019). To quantify which bee species showed increasing or decreasing abundance along the entire elevational gradient, we conducted Titan Indicator Taxon for all taxa that occurred in three or more sites in the R package “TITAN2” (Baker et al. 2015). To quantify which bee species had statistically significant associations with each or combinations of the categorical elevational bands, we used the R package “indicspecies” (de Cáceres and Legendre 2009).

To evaluate network indices along the elevational gradient, we constructed a quantitative interaction matrix of all observed bee–plant interactions for each plot across the four censuses, resulting in 28 weighted bee–plant networks. A priori planned contrasts that tested for a steady increasing or decreasing linear trend in the network indices were constructed using the following mathematical formula:

$$L=-2\left(\mu_{\mathrm{low}}\right)-1\left(\mu_{\mathrm{mid}-\mathrm{low}}\right)+0\left(\mu_{\mathrm{mid}}\right)+1\left(\mu_{\mathrm{mid}-\text{high}}\right)+2\left(\mu_{\text{high}}\right.)$$

Contrasts were conducted using the R package “multcomp” (Hothorn et al. 2008). Planned contrasts are used to provide extra power when detecting specified deviations (Kaltenbach 2021).

Network indices can be affected by the observed number of interactions in the network (Schleuning et al. 2012). To take into consideration the network size of each network and to ensure that the values could be compared to each other, all metrics were standardized using null models. The Patefield null‐model algorithm (r2d method) was used to generate 1000 permutations for each network metric. The r2d function uses the Patefield algorithm to generate random contingency tables with given marginals while maintaining the sum of the rows and columns. Averages of the null model scores were then subtracted from the observed values to obtain the ΔH2′, ΔWeighted Connectance, ΔQ, and ΔNODF.

## 3 Results

A total of 5242 bee interactions representing 157 bee species were observed across the elevational gradient during the study (Table 1). Sample coverage was 98.44% at the low (L) elevational band, 98.26% at the low‐mid (LM) elevational band, 97.10% at the mid (M) elevational band, 97.53% at the mid‐high (MH) elevational band, and 98.46% at the high (H) elevational band. Regardless of the method used, estimates of bee species richness in the mid (M) elevational band were significantly higher compared to all other elevations (Figure 3). The low (L) elevational band had a statistically significant lower number of bee species compared to all the other elevational bands, according to bee species richness rarefied by abundance (Figure 3C), while the ACE abundance‐based species richness estimator estimated bee richness to be statistically lowest at the highest elevational band (Figure 3A).

**TABLE 1:** Bee species collected along the elevational gradient at 5 elevational bands.

Taxon	Indicator species	Atitudes (m)					
	Linear/Categorical	L	LM	M	MH	H	Total
**Apidae**							
**Apinae**							
*Apis mellifera* (Linneaus, 1758)		86	73	79	77	78	393
*Bombus pullatus* (Franklin, 1913)		0	0	3	8	4	15
*Bombus volucelloides* (Gribodo, 1892)		0	0	0	0	29	29
*Centris analis* (Fabricius, 1804)		0	2	0	0	0	2
*Centris dichrootricha* (Moure, 1945)		0	0	4	0	0	4
*Centris nitida* (Smith, 1874)		0	0	4	0	0	4
*Centris obscurior* (Michener 1954)		1	0	0	0	0	1
*Centris terminata* (Smith, 1874)	/(M)	0	2	15	4	0	21
*Centris varia* (Erichson, 1849)		3	0	2	0	0	5
*Epicharis lunulata* (Mocsary, 1898)	/(LM)	0	6	0	0	0	6
*Epicharis rustica* (Olivier, 1789)		0	0	1	0	0	1
*Cephalotrigona zexmeniae* (Cockerell, 1912)		0	7	1	0	0	8
*Dolichotrigona schulthessi* (Friese, 1900)		10	2	0	0	0	12
*Freseomelitta paupera* (Provancher, 1888)		1	0	0	0	0	1
*Geotrigona lutzi* (Camargo & Moure, 1996)		1	1	1	0	0	3
*Melipona panamica* (Cockerell, 1912)		0	0	1	5	4	10
*Nannotrigona mellaria* (Smith, 1862)		0	2	3	0	0	5
*Nannotrigona perilampoides* (Cresson, 1878)	(Decr)/	43	36	3	0	0	82
*Oxytrigona mellicolor* (Packard, 1869)	/(L)	41	0	0	0	0	41
*Partamona orizabaensis* (Strand, 1919)		18	15	53	56	58	200
*Plebeia frontalis* (Friese, 1911)	(Decr)/(L)	33	9	5	2	0	49
*Plebeia jatiformes* (Cockerell, 1912)		0	0	4	0	0	4
*Plebeia llorentei* (Ayala, 1999)		2	0	0	0	0	2
*Plebeia pulchra* (Ayala, 1999)		12	1	21	12	0	46
*Plebeia tica* (Wille, 1969)		1	0	0	0	0	1
*Scaptotrigona subobscuripennis* (Shwarz, 1951)	(Incr)/	0	0	0	44	14	58
*Scaptotrigona pectoralis* (Dalla Torre, 1896)		0	1	1	0	0	2
*Scaura argyrea* (Cockerell, 1912)	/(L)	4	1	0	0	0	5
*Tetragona perangulata* (Cockerell, 1917)		12	12	0	0	0	24
*Tetragona ziegleri* (Friese, 1900)	(Decr)/	20	22	29	13	0	84
*Tetragonisca angustula* (Latreille, 1811)	(Decr)/	53	79	82	17	24	255
*Trigona corvina* (Cockerell, 1913)	(Decr)/	125	362	140	1	0	628
*Trigona fulviventris* (Guerin, 1844)	(Incr)/(H + MH)	139	107	131	396	426	1199
*Trigona fuscipennis* (Friese, 1900)	(Decr)/(L + LM)	103	115	0	0	0	218
*Trigona silvestriana* (Vachal, 1908)	(Decr)/	137	129	93	0	0	359
*Trigonisca pipioli* (Ayala, 1999)		81	7	13	0	0	101
*Eufriesea schmidtiana* (Friese, 1925)		0	0	1	0	0	1
*Euglossa allosticta* (Moure, 1969)		1	0	0	0	0	1
*Euglossa azureoviridis* (Friese, 1930)		0	1	1	0	0	2
*Euglossa bursigera* (Moure, 1970)		0	0	1	0	0	1
*Euglossa cyanura* (Cockerell, 1917)	/(H)	0	2	1	3	19	25
*Euglossa cybelia* (Moure, 1968)		0	0	0	0	1	1
*Euglossa deceptrix* (Moure, 1968)		0	1	1	0	0	2
*Euglossa maculilabris* (Moure, 1968)	/(H)	0	0	0	2	10	12
*Euglossa townsendi* (Cockerell, 1904)		2	3	3	2	0	10
*Euglossa tridentata* (Moure, 1970)		1	1	0	0	1	3
*Euglossa variabilis* (Friese, 1899)		0	1	1	0	0	2
*Euglossa viridissima* (Friese, 1899)		0	14	7	0	2	23
*Eulaema polychroma* (Mocsary, 1897)		0	0	0	0	1	1
*Eulaema luteola* (Moure, 1967)		0	0	0	0	2	2
*Eulaema speciosa* (Mocsary, 1897)		0	0	0	5	5	10
*Exarete smaragdina* (Guerin, 1844)		1	0	0	0	0	1
*Exomalopsis* sp.2		2	0	3	0	0	5
*Exomalopsis* sp.3		0	0	0	2	3	5
*Exomalopsis* sp.4		0	1	15	2	7	25
*Exomalopsis* sp.7		0	0	1	2	0	3
*Melitoma* sp.1		0	0	1	0	0	1
*Melitoma* sp.2		0	0	1	0	0	1
**Eucerinae**							
*Lophopedia apicalis* (Cresson, 1878)		0	1	0	0	0	1
*Lophopedia klugi* (Friese, 1899)		0	0	1	3	0	4
*Lophopedia pygmaea* (Schrottky, 1902)		0	0	1	0	0	1
*Lophopedia* sp.2		0	5	0	0	0	5
*Nasutopedia morena* (Aguiar, 2018)		0	0	0	0	5	5
*Paratetrapedia albilabris* (Friese, 1917)		0	0	6	1	0	7
*Paratetrapedia calcarata* (Cresson, 1878)	/(M)	5	12	49	11	1	78
*Paratetrapedia connexa* (Vachal, 1909)		0	5	0	0	0	5
*Paratetrapedia lugubris* (Cresson, 1878)		0	19	4	0	0	23
*Paratetrapedia moesta* (Cresson, 1878)	/(L)	3	0	0	0	0	3
*Melissodes* sp.1		0	1	0	0	0	1
*Peponapis utahensis* (Cockerell, 1905)		0	0	1	0	0	1
*Thygater aethiops* (Smith, 1854)	/(H)	0	0	0	0	25	25
*Thygater* sp.1		0	0	0	1	6	7
*Thygater* sp.2	/(H)	0	0	0	0	6	6
*Thygater* sp.3		0	2	2	2	0	6
**Xylocopinae**							
*Ceratina buscki* (Cockerell, 1919)	(Decr)/	3	6	4	0	0	13
*Ceratina chloris* (Fabricius, 1804)		0	0	1	0	0	1
*Ceratina cobaltina* (Cresson, 1878)	(Decr)/(L + LM)	10	11	3	0	0	24
*Ceratina dimidata* (Friese, 1910)		0	0	1	1	5	7
*Ceratina eximia* (Smith, 1862)	/(L + M)	31	1	23	0	0	55
*Ceratina ignara* (Cresson, 1878)		0	1	1	1	1	4
*Ceratina rectangulifera* (Schwarz & Michener, 1954)	(Decr)/	61	52	40	1	1	155
*Ceratina trimaculata* (Friese, 1916)	(Incr)/	0	0	8	16	6	30
*Ceratina zeteki* (Cockerell, 1934)		0	0	0	0	13	13
*Ceratina* sp.1	(Decr)/(L)	4	2	0	0	0	6
*Ceratina* sp.2		1	8	0	0	0	9
*Ceratina* sp.4	(Decr)/	8	10	3	0	0	21
*Ceratina* sp.5		0	1	0	0	0	1
*Ceratina* sp.6		0	0	2	0	0	2
*Xylocopa fimbriata* (Fabricius, 1804)		0	0	1	0	0	1
*Xylocopa gualanensis* (Cockerell, 1904)		0	2	0	0	0	2
*Tetrapedia maura* (Cresson, 1878)		0	1	0	0	0	1
**Halictidae**							
**Halictinae**							
*Agapostemon leunculus* (Vachal, 1903)		0	0	0	1	0	1
*Agapostemon nasutus* (Smith, 1853)		0	0	14	0	0	14
*Augochlora (Augochlora) albiceps* (Friese, 1925)		1	2	0	0	0	3
*Augochlora (Oxystoglossella) aurifera* (Cockerell, 1897)		3	0	0	1	2	6
*Augochlora (Oxystoglossella) cardiaefloris* (Cockerell, 1907)	(Decr)/(L+LM+M)	14	25	30	2	0	71
*Augochlora (Augochlora) nigrocyanea* (Cockerell, 1897)	(Decr)/(L)	44	11	4	0	0	59
*Augochlora (Oxystoglossella) nominata* (Michener, 1954)		0	6	7	3	1	17
*Augochlora (Augochlora) obscuriceps* (Friese, 1925)		0	0	1	0	0	1
*Augochlora (Augochlora) quiriguensis* (Cockerell, 1913)		3	0	4	1	0	8
*Augochlora (Augochlora) sidafoliae* (Cockerell, 1913)		0	0	2	0	0	2
*Augochlora (Augochlora) smaragdina* (Friese, 1916)		0	1	1	3	7	12
*Augochlora* (Augochlora) sp.4		1	0	0	0	0	1
*Augochlorella comis* (Vachal, 1911)	/(LM)	0	4	0	0	0	4
*Augochlorella neglectula* (Cockerell, 1897)	(Decr)	1	14	23	0	0	38
*Augochlorella pomoniella* (Cockerell, 1915)		0	1	5	0	0	6
*Augochloropsis auriventris* (Friese, 1921)		0	0	1	0	0	1
*Augochloropsis ignita* (Smith, 1861)		1	2	2	0	0	5
*Augochloropsis metallica* (Fabricius, 1793)		0	2	3	0	0	5
*Caenaugochlora (Caenaugochlora)* sp.1		0	0	0	0	3	3
*Caenaugochlora (Caenaugochlora) gemmella* (Cockerell, 1912)		0	0	1	1	0	2
*Caenaugochlora (Caenaugochlora)* sp.2		0	0	1	0	0	1
*Lasioglossum (Dialictus) cupreicollis* (Friese, 1916)	/(MH)	0	0	11	41	1	53
*Lasioglossum* sp.1		0	0	1	3	4	8
*Lasioglossum (Eickwortia) alexanderi* (McGinley, 1999)		0	0	0	2	8	10
*Lasioglossum (Dialictus)* sp.1	(Incr)/(H)	1	8	11	6	24	50
*Lasioglossum (Dialictus)* sp.2	(Incr)/(H)	0	0	2	4	14	20
*Lasioglossum (Dialictus)* sp.3		0	0	0	1	3	4
*Lasioglossum (Dialictus)* sp.4	/(MH)	0	0	1	11	1	13
*Lasioglossum (Dialictus)* sp.5	(Incr)/(H)	0	0	0	3	7	10
*Lasioglossum (Dialictus)* sp.7		0	0	0	3	2	5
*Lasioglossum (Dialictus)* sp.8		18	23	21	9	15	86
*Lasioglossum (Dialictus)* sp.9		0	0	0	1	0	1
*Lasioglossum (Dialictus)* sp.10		0	0	0	0	1	1
*Lasioglossum (Dialictus)* sp.11		2	0	0	0	0	2
*Lasioglossum (Dialictus)* sp.12		1	1	0	0	0	2
*Lasioglossum (Dialictus)* sp.13		0	1	0	1	0	2
*Lasioglossum (Dialictus*) sp.14		0	0	0	2	4	6
*Lasioglossum (Dialictus) tessellatosum* (Gardner and Gibbs 2020)	/(LM+M)	0	2	3	0	0	5
*Habralictus* sp.2		0	0	0	2	0	2
*Halictus hesperus* (Smith, 1862)		1	0	0	0	0	0
*Halictus (Halictus) ligatus* (Say, 1837)		0	1	2	3	0	6
*Neocorynura discolorata* (Smith‐Pardo, 2005)		0	0	0	1	1	2
*Neocorynura lignys* (Vachal, 1904)		0	0	0	0	1	1
*Neocorynura pubescens* (Friese, 1916)	/(H)	0	0	0	0	4	4
*Neocorynura centroamericana* (Smith‐Pardo, 2005)		0	0	0	2	0	2
*Neocorynura rufa* (Michener, 1954)		0	0	0	0	2	2
*Pseudaugochlora graminea* (Fabricius, 1804)	(Incr)/	0	0	6	5	8	19
*Pseudaugochlora sordicutis* (Vachal, 1904)	/(H)	0	0	0	1	4	5
**Megachilidae**							
**Megachilinae**							
*Anthodioctis* sp.1		0	0	2	0	0	2
*Anthodioctes* sp.3		0	0	0	1	4	5
*Coelioxys* sp.1		0	0	1	0	0	1
*Coelioxys* sp.3		1	0	0	0	0	1
*Dolichostelis costaricensis* (Friese, 1921)		0	2	0	0	0	2
*Hypanthidium melanopterum* (Cockerell, 1917)		1	0	0	0	0	1
*Megachile* sp.2		0	0	1	1	1	3
*Megachile* sp.3		0	0	11	0	0	11
*Megachile* sp.4		0	1	0	0	0	1
*Megachile* sp.5		0	0	0	1	0	1
*Megachile* sp.7		0	0	0	2	0	2
*Megachile* sp.8		0	0	0	1	1	2
*Megachile* sp.9		1	0	0	1	0	2
*Megachile* sp.10		0	0	1	1	1	3
*Megachile* sp.11		0	0	0	0	4	4
*Megachile* sp.12		0	0	0	1	0	1
**Colletidae**							
*Chilicola ashmeadi* (Crawford, 1906)	(Incr)/	0	0	15	32	25	72
*Hylaeus* sp.2		0	1	2	4	0	7
Total number of bee species		52	67	85	64	56	
Total number of plant species		42	47	65	46	65	
Total number of links		1153	1263	1067	850	909	
Number of unique species		13	11	21	7	15	

*Note:* Species highlighted were only collected from one elevational band as follows: Vermillion (L); orange (LM); yellow (M); teal green (MH); sky blue (H).

**FIGURE 3:**
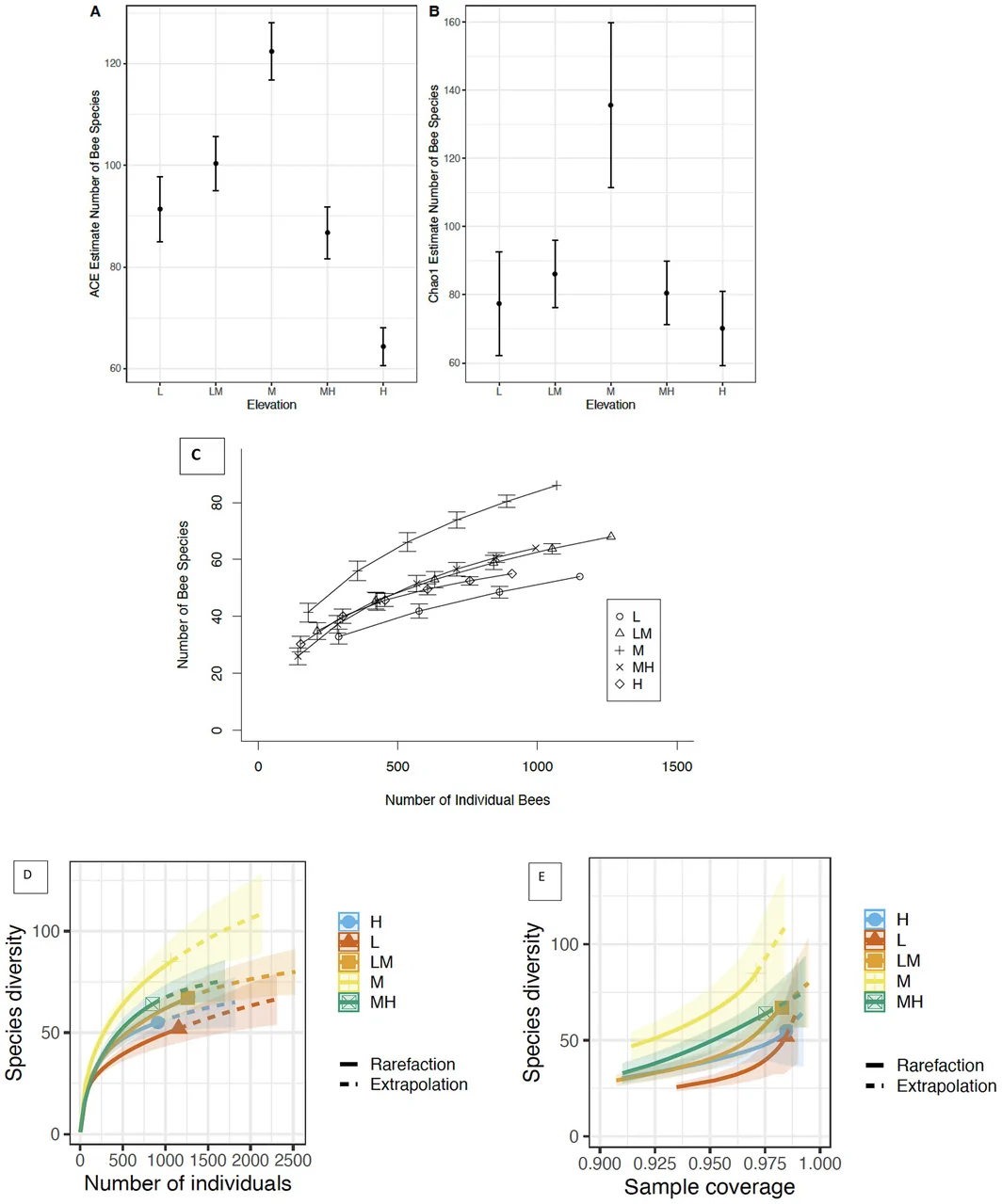
(A) Abundance‐based ACE Estimates of bee species richness per elevational band, where L is 200–300 m elevation, LM is 500–600 m elevation, M is 800–900 m elevation, MH is 1100–1200 m elevation, and H is 1400–1500 m elevation. (B) Abundance‐based Chao1 Estimates of bee species richness per elevational band. (C) Rarefied bee species richness by elevational band. Species richness was rarefied by abundance of bees collected from each elevation band. Vertical lines at the intervals represent the standard error. (D) Bee species richness estimates and 95% CI by elevation band using sample‐sized‐based rarefaction and extrapolation sampling curves. (E) Bee species richness estimates and 95% CI by elevational band using coverage‐based rarefaction and extrapolation sampling curves.

Of the 157 bee species, 57 were represented by fewer than 3 individuals and an additional 19 were collected from fewer than 3 sites, and these species were excluded from the indicator analysis. Of the 81 remaining species, 23 met the criteria for decreasing (15 species) or increasing (8 species) abundance along the elevational gradient (Table 1). Indicator analysis of bee associations with different elevation bands found that nine bee species were statistically associated with the high elevation band (H); six species were associated with the low elevation band (L); for the remaining elevational bands, low‐mid, mid, and mid‐high, each band had two bee species that were significantly associated (Table 1). Six bee species had significant relationships with two or more elevational bands; for example, the stingless bee species *Trigona fulviventris* was statistically more abundant at the H and MH elevational bands, while the stingless bee species *Trigona fuscipennis* was statistically more abundant in the L and LM elevational bands (Table 1).

There was a statistically significant difference in bee communities between the elevational bands (ANOSIM R statistic 0.65; *p* = 0.001). The dissimilarity in bee species composition along the entire elevational gradient measured as Bray–Curtis dissimilarity was 0.675. This value was partitioned into two components to determine how much change in bee community composition along the elevational gradient was due to species replacement versus species loss or nestedness. Species replacement measured as balanced abundance was the dominant phenomenon, explaining 93% (0.628 of 0.675) of community dissimilarity, while nestedness measured as abundance gradient explained 7% (0.047 of 0.675). The elevational bands MH and H were statistically more similar to each other than the other three elevational bands, with the L elevational band marginally statistically different from the elevational bands LM and M, which clustered together (Figure 4, Figure S1).

**FIGURE 4:**
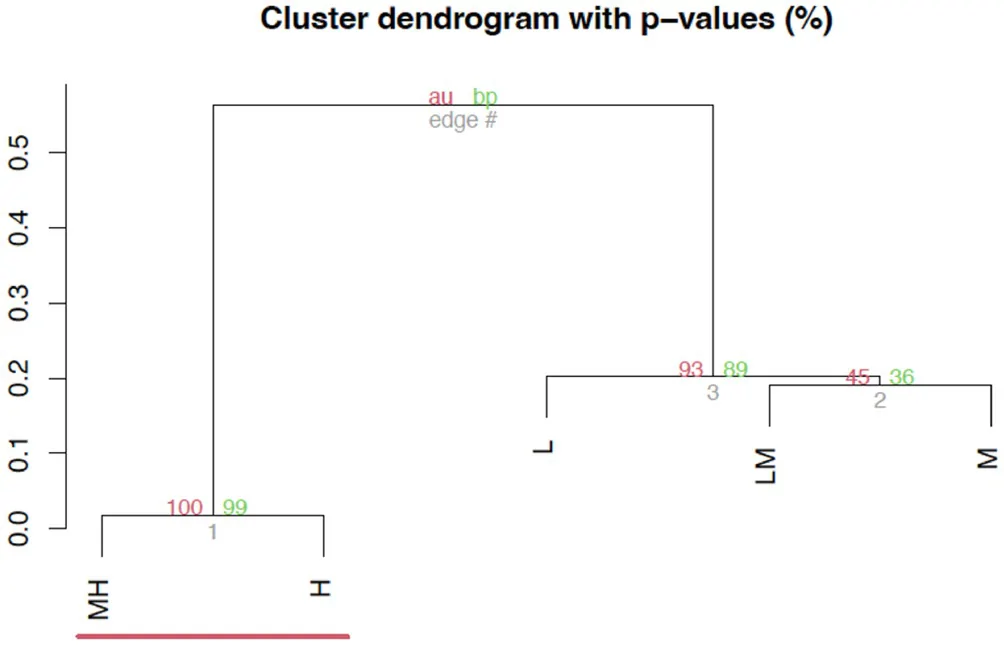
Cluster diagram of bee community similarity by elevational band, where L is 200–300 m elevation, LM is 500–600 m elevation, M is 800–900 m elevation, MH is 1100–1200 m elevation, and H is 1400–1500 m elevation.

A total of 165 plant species accounting for 152 genera were observed across the elevational gradient (Table S3). The plant species with the most observed interactions were *Musa* spp. (422; 8%), *Caesalpinia pulcherrima* (272; 5%), *Portulaca* spp. (248; 4.8%), *Cosmos sulphureus* (212; 4%) and *Turnera subulata* (212; 4%). Plant species richness ranged from 6 to 26 across the 28 plots. Plant species richness was not statistically different between the elevational bands (*F*_4,23_ = 0.703; *p* = 0.598; Figure S2).

All empirical networks were significantly more specialized (*Z* = 4.05–42.08), more modular (*Z* = 8.12–33.94), and less connected (*Z* = −2.32 to −16.52) than expected by chance (Table 2; Table S4). All networks except two were significantly less nested than expected by chance (*Z* = −0.62 to −8.85; Table 2; Table S4). Network specialization (H2′) significantly decreased with increasing elevation (*t*_23_ = −7.03, *p* < 0.001; Figure 5A). Connectance increased with increasing elevation (*t*_23_ = 4.41, *p* < 0.001; Figure 5B). Modularity decreased as elevation increased (*t*_23_ = −6.103, *p* < 0.001; Figure 5C). Nestedness also increased with increasing elevation (*t*_23_ = 4.35, *p* < 0.001; Figure 5D).

**TABLE 2:** Network metrics of 28 bee–plant networks along a Neotropical elevational gradient’.

Plot	Elevational band	H2′	Connectance	*Q*	NODF	Bee species	Plant species	Links	H2′ Z‐score	Connectance Z‐score	Q Z‐score	NODF Z‐score
Guillermo	L	0.7339	0.0483	0.6759	6.409	31	22	271	34.056	−16.519	33.950	−7.328
LilaCruz	L	0.5609	0.0694	0.5869	12.838	33	22	347	42.0818	−22.89	32.725	−7.577
LupesMom	L	0.6357	0.068	0.5165	13.006	29	15	348	32.5	−20.038	30.582	−8.718
MRamirez	L	0.6108	0.0809	0.6045	10.27	21	21	187	23.292	−11.714	23.660	−4.857
Dimas	LM	0.6216	0.0923	0.5798	9.65	22	11	156	19.861	−9.894	21.148	−5.013
Aristetes	LM	0.5619	0.0468	0.4244	8.9316	46	26	529	26.367	−22.855	28.732	−8.855
Josefina	LM	0.5736	0.086	0.5245	11.697	35	14	220	21.676	−15.162	18.458	−6.765
Lucy	LM	0.5675	0.1059	0.5981	7.425	16	14	93	12.431	−6.494	13.342	−4.525
Oscar	LM	0.6827	0.0703	0.6799	8.9797	24	16	176	29.494	−15.833	25.491	−6.114
Pipe	LM	0.7285	0.1153	0.528	21.148	14	6	89	12.147	−7.911	14.876	−3.205
Blanca	M	0.6932	0.0583	0.6078	6.928	28	23	208	21.943	−13.1	24.123	−6.122
Memei	M	0.4612	0.0916	0.5934	13.454	28	23	137	17.725	−5.286	13.021	−0.623
Ramon	M	0.7623	0.0807	0.666	9.03	33	13	210	26.895	−17.561	25.910	−8.205
Victor	M	0.4958	0.0940	0.5365	8.74	30	14	144	14.891	−8.019	13.279	−4.736
Yolanda	M	0.5948	0.0788	0.5023	10.038	35	13	183	12.835	−9.775	15.046	−5.337
Zulay	M	0.4678	0.0871	0.569	11.516	30	24	185	20.201	−6.943	17.247	−2.848
Carlos	MH	0.5131	0.126	0.525	14.42	15	9	144	20.980	−12.357	17.429	−6.367
Danilo	MH	0.7816	0.0799	0.6053	8.857	16	11	84	10.444	−8.725	14.142	−4.017
Elisa	MH	0.4698	0.104	0.5853	13.444	16	14	65	10.587	−4.857	8.120	−1.803
Maco	MH	0.4622	0.0961	0.3426	13.729	22	23	175	5.738	−6.28	9.081	−4.030
Oldemar	MH	0.5621	0.1042	0.408	15.751	23	24	229	10.798	−10.56	15.580	−4.031
Sergio	MH	0.3486	0.1215	0.4939	15.26	27	14	128	11.639	−2.315	10.864	−2.393
Belmar	H	0.5696	0.0968	0.2597	13.18	18	15	192	4.047	−9.4	10.621	−5.419
ElBosque	H	0.409	0.108	0.5027	12.79	32	13	130	11.346	−3.867	10.127	−2.507
Mariposa	H	0.5999	0.1154	0.456	5.935	17	25	138	8.083	−6.813	13.497	−5.512
Mayra	H	0.4783	0.0828	0.5194	11.268	29	24	194	18.653	−8.982	14.629	−4.703
Peter	H	0.5143	0.110	0.404	16.128	18	10	113	8.438	−6.568	9.724	−2.961
Tercicio	H	0.4503	0.0962	0.474	10.723	27	20	142	11.538	−5.32	9.131	−2.950

*Note:* Empirical values and standardized z‐scores from 1000 null models are shown for Specialization (H2′), weighted connectance, modularity Q and Nestedness, represented as NODF (Nestedness Overlap and Decreasing Fills).

**FIGURE 5:**
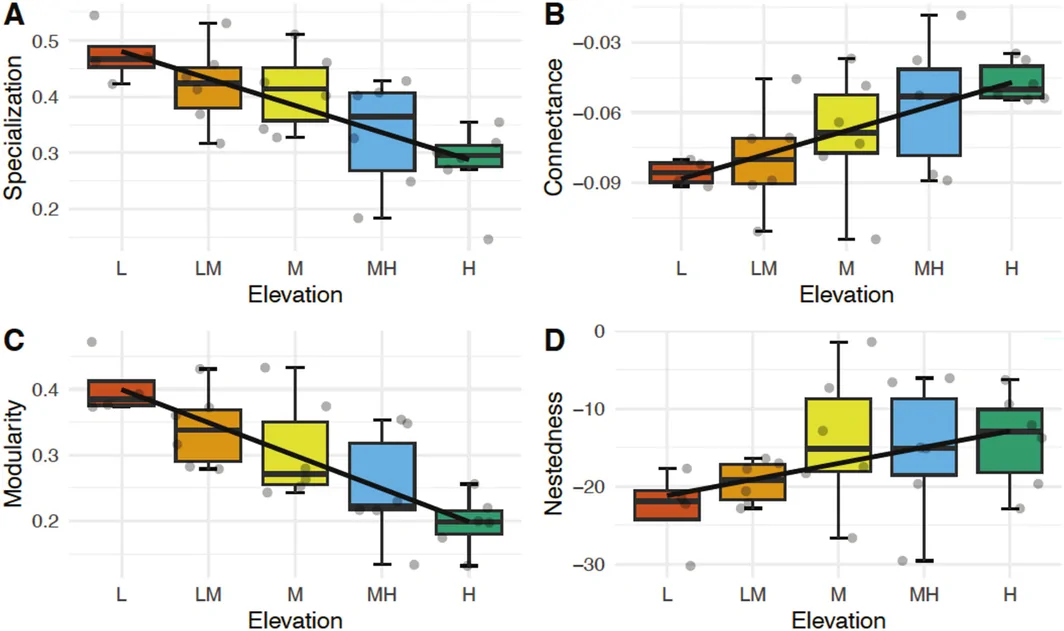
Mean and standard error of (A) the index of specialization, (B) connectance, (C) modularity (Q), and (D) nestedness with increasing elevation where L is 200–300 m elevation, LM is 500–600 m elevation, M is 800–900 m elevation, MH is 1100–1200 m elevation, and H is 1400–1500 m elevation. Gray points represent the Δ value of the network value at each plot in the elevational band. The black trend line indicates the overall direction of the trend.

## 4 Discussion

Bee species richness and bee–plant network indices were found to follow distinct patterns, where the number of bee species peaked at middle elevations, specialization and modularity both decreased linearly with elevation, and connectance and nestedness both increased linearly from low to high elevations. This study represents the first elevational gradient study of Neotropical bee–plant ecological interaction networks that includes elevations below 3000 m, as well as includes replicate plots along a single elevational transect. The bee community along the elevational gradient was characterized by high species replacement, many species with elevational associations or unique to elevational bands, and many species with low detections.

Of the five tropical studies quantifying the relationships between bee–plant ecological interaction network indices and elevation, two were Paleotropical (Classen et al. 2020; Dzekashu et al. 2023), and three were Neotropical (Moreira et al. 2020; Pelayo et al. 2021; Jacob Cristóbal‐Perez et al. 2024). Within the three Neotropical studies, two were carried out at elevations above 3000 m (Pelayo et al. 2021, Jacob Cristóbal‐Perez et al. 2024), while the third was a meta‐analysis, with conclusions regarding the relationships between altitude and network indices obtained from bee–plant networks within the tropics as well as outside the tropics (Moreira et al. 2020). For specialization, H2′, our findings lend support for the most common relationship across these five tropical studies, that of decreasing specialization with altitude (Classen et al. 2020; Jacob Cristóbal‐Perez et al. 2024), and contrasts the finding of no effect of elevation (Dzekashu et al. 2023). Broadly, specialization decreasing with elevation has also been documented outside the tropics (Hoiss et al. 2015; Lara‐Romero et al. 2019; Chesshire et al. 2021). Just as many recent studies outside of the tropics, however, have found opposing relationships between specialization and elevation, including increasing (Wang et al. 2024), oscillating (Aguirre and Junker 2024), and no change (Adedoja et al. 2018) with increasing elevation. These variable results may be explained by the relative abundance of different insect pollinator taxa with varying degrees of specialization in the different locations studied, as (Dzekashu et al. 2023) was the only one other study besides ours that evaluated only the bee community. However, even within the bee community, relative abundance of different bee species can also shape results, as (Dzekashu et al. 2023) found no change in specialization over the elevational gradient, but 81% of interactions were of the western honeybee, *Apis mellifera* , for which we found no relationship between relative abundance and elevation of this bee species, and our bee community was dominated by a diverse suite of stingless bees (65%) that showed strong relationships with elevation (Table 1). Furthermore, in our study *A. mellifera* comprised only 7% of observations and the most abundant bee in our study, *T. fulviventris* , comprised only 23% of observations and was found by both indicator analyses to be strongly associated with higher elevations. Similar to latitudinal gradients of specialization, results of altitudinal gradients of specialization may depend on the taxa and types of interactions studied, the range of elevations included, as well as the specialization measure used to compare networks (Pinheiro et al. 2023). With many elevational gradient studies finding strong relationships between elevation and specialization, it is also important to note that future studies examining latitudinal gradients of specialization should ensure that the locations compared are of similar altitude, as well as from the mainland.

Connectance is known to decrease with the range of possible interactions or increasing species richness (Olesen et al. 2007; Vizentin‐Bugoni et al. 2018; Dzekashu et al. 2023), yet after accounting for network size, the pattern of connectance along the elevational gradient did not correspond to the elevational pattern of bee species richness we found. One plausible explanation for the higher connectance at higher elevations is that high and mid‐high elevation networks were dominated by the super‐generalist bee species, *T. fulviventris* (47% at both MH and H), while all lower elevation networks had a much lower proportion of this species comprising the interactions (8%–12% at L, LM and M). Temperature may be a driving force in both bee diversity (Classen et al. 2015) and network indices (Classen et al. 2020). Lower mean annual temperatures at high elevations may require more energy expenditure for ectothermic pollinators to maintain their activities and many bee species reduce overall foraging efforts (Dellinger et al. 2021). In some cases, low temperatures are expected to lead to more specialized interactions and lower connectance because at the highest elevations, interspecific competition is reduced leading to niche expansion (Valdovinos et al. 2016). Due to the unpredictable cold and wet conditions of a montane forest, plants at higher elevations have also adopted self‐compatibility for pollination (Bawa 1990), which typically leads to lower connectance values at high elevations (Pelayo et al. 2021). Our study was conducted on a short tropical mountain (peak of 1800 m), which is not uncommon in the tropics, and our results could be attributed to both the lack of elevations over 1800 m, and the fine‐scale resolution of elevational bands studied, that is, with every 200 m elevational change.

Considering that altitudes in the study area reached as high as ca. 1550 m.asl., our results indicate a peak in bee richness at intermediate altitudes, a pattern that has been documented for Paleotropical bee communities (Widhiono et al. 2017). This pattern may result from the overlapping ranges of species adapted to high‐ or low‐altitude environments co‐occurring in the mixed environmental conditions of intermediate elevations (Colwell et al. 2004). Higher species richness at intermediate altitudes is frequently observed in tropical communities and has been documented for the bird community of Pacific Slope Mesoamerica (Jankowski et al. 2009). Mid‐elevation and low‐elevation peaks have been observed in tropical insects, including both patterns from the much higher mountain encompassing the Barva Transect in Costa Rica (Colwell et al. 2008; Kumar et al. 2009). For the overall pollinator community, low‐elevation peaks (Lara‐Romero et al. 2019; Moreira et al. 2020; Pelayo et al. 2021; Jacob Cristóbal‐Perez et al. 2024), mid‐elevation peaks (Adedoja et al. 2018; Chesshire et al. 2021), and seasonal changes in low‐ and mid‐elevation peaks (Dzekashu et al. 2023). Mixed results for the overall pollinator community may be due to the inclusion of taxa that respond differently to elevation, as (Wang et al. 2024), found that Hymenoptera richness decreased while Diptera richness increased with increasing elevation. Similarly, shifts from bee‐dominated pollination at lower elevations to fly‐dominated pollination at higher elevations have been reported (McCabe et al. 2019), which could also affect network metrics in these studies.

Quantifying the diversity in functional traits of the bee community could resolve the differences we found between network indices and diversity patterns, as recent work in alpine systems finds strong relationships between the network indices we evaluated and pollinator functional trait diversity (Aguirre and Junker 2024). This may imply that the bee communities we studied are more functionally diverse at lower elevations, with the highest functional redundancy at middle elevations. One question that arises is, if bee functional diversity patterns differ from bee species richness patterns along the elevational gradient, in contrast to alpine systems, could this be explained by the presence of ecologically similar clusters of bee species at tropical mid‐altitudes, similar to the phenomenon of “species swarms” described for some tropical plant genera (Sedio et al. 2012). Other studies have shown higher functional overlap at higher elevations due to stressful environmental conditions filtering species with similar traits that are best adapted to the harsh conditions (Classen et al. 2017; Lara‐Romero et al. 2019).

The bee community we studied showed substantial turnover between elevational bands, with 95% of beta‐diversity attributed to species replacement. High turnover in plant and bird communities have been documented for the seasonally dry Pacific slopes of Mesoamerica, where well‐recognized gradients of moisture, temperature and seasonality exist (Jankowski et al. 2009). This finding implies that the bee species we studied have narrow local distributions. Tropical insects are known to have more narrow thermal tolerances compared to their temperate counterparts (Deutsch et al. 2008), and the finding that birds and plants also display narrow local distributions in the seasonally dry tropics highlights the extreme vulnerability of insect communities in these ecosystems to both temperature and precipitation changes caused by deforestation and climate change (Mccain and Colwell 2011). Indeed, some stingless bee species' altitudinal distributions that have been documented previously in the region (Ortiz‐Mora et al. 1995; Conrad et al. 2021) are now being collected from higher elevations. During the year of our study, 2023, an extreme delay in the onset of the rainy season led to a severe drought in the study area during the months of April and May, and this drought had the potential to influence the results of our study. In the tropics, the drivers of plant phenology are less understood compared to temperate and subtropical biomes, but plant phenology is known to respond to global precipitation patterns produced by ENSO cycles that may or may not be greatly altered by climate change (Ramaswami et al. 2019). Although speculative, droughts such as the one we experienced during the 2023 field season seem to be more common and may impact the bee community in a variety of ways, for example, causing delays in bee emergence and peak flowering times, decoupling of interactions, or increased bee mortality due to desiccation or inability to provision new cells for offspring. Bee species that don't live in perennial colonies in the seasonally dry tropics may be less robust to the changing seasonality in rainfall (Conrad et al. 2021), and we observed many charismatic species to be seemingly more absent from the bee community compared to our 2022 field season with a more normal rainy season, for example, 68 individuals in 2022 versus 11 individual in 2023 of 3 Augochloropsis species, despite the much broader sampling of elevations in 2023. While seasonal patterns of precipitation continue to become less predictable in the tropics, insect emergence cues, flowering phenology cues, and plant response to climate change are still poorly understood in the tropics (Wright and Calderón 2018; Ramaswami et al. 2019). The ecological impacts from either loss of the interactions or the species themselves due to land use change or climate change points toward the need to conduct more field studies such as this one that can help parameterize predictive models of plant–pollinator interactions (Peralta et al. 2024).

Our study indicates that the successful conservation of tropical bees will require action across many different elevations in tropical mountains. For example, we identified a unique community of high‐elevation mountain‐dwelling bee species. We expected bee species richness would continue to decrease as elevation increased based on previous studies conducted on tropical mountains (Perillo et al. 2017; Dellinger et al. 2021; Dzekashu et al. 2023), and from previous work surveying bee communities at elevations from 800 to 1100 m (Conrad et al. 2021). Surprisingly, at the highest (H) elevational band, 1400–1500 m, the number of bee species was still very high, and indicator analysis found the highest number of species significantly associated with this elevational band compared to all other bands (nine species). In addition, because indicator analyses requires species to have been collected from at least three plots, the following species could not be statistically analyzed with indicator analyses; however, it is likely owing to data from other years in the study area that the following species are only found in, or strongly associated with, the high elevational band: *Bombus volucelloides* (29 individuals), *Ceratina zeteki* (13 individuals), *Nasutopedia morena* (4 individuals), and 3 *Eulema* species (13 individuals) among others (Table 1). It is important to note that the mountain on which this study was conducted is part of the Tilarán mountain range, and this mountain range, as well as the adjacent Guanacaste mountain range, have much lower highest peaks compared to the Central Volcanic and Talamanca mountain ranges, ranging from 1500 to 2000 m elevation, as well as much lower amounts of available highland habitat (Barrantes 2009). This means that as the climate warms, the unique community of bees living at 1400–1500 m elevation in these two mountain ranges may not have available upslope habitat and is therefore potentially at high risk of local extirpation. Asymmetric aggression could also be particularly important. For example, if warming in montane landscapes allows upslope range expansion by dominant competitors, then high‐elevation subordinate species could be forced into progressively smaller mountaintop habitats, jeopardizing the viability of their populations (Jankowski et al. 2010).

At low elevations, the bee community is highly specialized and modular, highlighting the vulnerability of this bee community as well as the pollination services they provide, where the loss of a few highly specialized or modular species can lead to a reduction in functional complementarity and potentially have a greater impact on pollination services (Blüthgen and Klein 2011) and ultimately farmer livelihoods here. Biotic attrition in tropical lowlands resulting from warming temperatures (Colwell et al. 2008) as well as the more severe drought that seasonally dry tropical forest lowlands experience could also heavily impact pollination services in tropical lowlands if no bee species are able to adapt to these climatic conditions. Finally, mid‐altitudes harbored the greatest richness of bee species, highlighting the high impact that conservation action at these elevations would have for bee communities, and the crops and terrestrial biodiversity they help generate and maintain (Balvanera et al. 2005; Fontaine et al. 2006). The tropical dry forest biome which encompassed elevations up to approximately 900 m (L, LM and M elevational bands) is an extremely endangered biome (Stan and Sanchez‐Azofeifa 2019; Alvarenga et al. 2020), and therefore it is important to identify which bee species are associated with this biome to ensure that conservation action supports these species. Previous studies have shown the importance of primary and late‐successional forest for eusocial bees in this biome (Alvarenga et al. 2020).

While we didn't sample from within the forest itself, most of the home gardens we sampled were in close proximity to late‐successional forest or forest patches; due to economic incentives of eco‐tourism in the area, there is higher forest cover. There is a potential that sampling in forests would have led to different conclusions; although eusocial Meliponine bees did dominate the bee community we studied (65%), other studies in forests of TDFs in Brazil found they represented 94% of the community and were more abundant in late‐successional forests (Alvarenga et al. 2020). Indeed, empirical datasets are lacking on the effects of landscape change and the loss of natural environments on network structure; however, some studies conducted have found that, in the tropics, native vegetation loss can lead to lower values of nestedness and specialization in human‐managed lands due to specialist species loss, or that the role of species changes from specialist to more generalist (Moreira et al. 2018). The plots we sampled in this study were embedded in landscapes with similar characteristics including large forest patches, pasture and other tropical countryside habitats, and therefore likely that if network structure differed in the plots we sampled from natural habitats, it would differ equally along the elevational gradient, however the possibility does exist that if natural habitat of higher elevations were originally comprised of more specialist species, landscape change could impact these elevations disproportionately compared to lower elevations. However, research to date indicates that loss of specialization would occur equally across elevations from natural to disturbed habitats, and therefore the observed pattern validly describes the elevational gradient. Finally, since pollination services are a critical ecosystem service for the people and wildlife who reside in tropical mountains, and these mountains are often mosaics of disturbed and natural habitat patches, it is important to quantify and understand these network metrics in both habitat types.

## Author Contributions

**Valerie E. Peters:** conceptualization (lead), data curation (equal), formal analysis (equal), funding acquisition (lead), investigation (equal), methodology (lead), project administration (lead), resources (lead), software (equal), supervision (lead), validation (lead), visualization (lead), writing – original draft (equal), writing – review and editing (lead). **Jenny Hsu:** data curation (equal), formal analysis (equal), investigation (equal), writing – original draft (equal).

## Funding

This work was supported by funding from the Earthwatch Institute and the Division of Environmental Biology (2142938) to V.E.P.

## Conflicts of Interest

The authors declare no conflicts of interest.

## Supporting information


**Table S1:** Elevational gradient pollination network studies quantifying differences in network indices by elevation. For each study we extracted the location, habitats sampled, latitude and longitude, taxonomic groups considered, elevational range, whether samples include replicates per elevation and the climate zone. Network property changes reflect trends in values as elevation increases.
**Table S2:** Definitions of network metrics mentioned in manuscript.
**Table S3:** Plant species recorded along elevational gradient. Plant species highlighted in gray were involved in more than 3% of bee–plant interactions observed along the elevational gradient.
**Table S4:** Bee–plant Network metrics full data including null model means and SD for each metric at each plot.
**Figure S1:** Distance‐based redundancy analysis ordispider grouped by home garden. Each black circle represents one of the home gardens. CAP1 represents elevation and increases as represented by mid‐high (1100–1200 m) and high‐elevation sites (1400–1500 m).
**Figure S2:** Mean ± SE of plant species richness in bipartite networks by elevational band.

## Data Availability

The data and code used for the analysis of this manuscript are available in a Dryad repository, which can be accessed through a private anonymous link created for the reviewing process (https://doi.org/10.5061/dryad.9kd51c5zd).
