# Supplementary material for: Network Indices and Diversity Follow Distinct Patterns for Bee–Plant Networks Along a Neotropical Elevational Gradient
Source: Ecol Evol. 2026 Oct 27;16(11):e74549. doi: 10.1002/ece3.74549 (PMC13713433; doi:10.1002/ece3.74549)
Supplement: Supplementary file 1 [file ECE3-16-e74549-s001.docx]

Appendices.

Table S1. Elevational gradient pollination network studies quantifying differences in network indices by elevation. For each study we extracted the location, habitats sampled, latitude and longitude, taxonomic groups considered, elevational range, whether samples include replicates per elevation and the climate zone. Network property changes reflect trends in values as elevation increases.

| Studies | Location | Habitat | Latitude | Longitude | Taxonomic Group | Elevation Range | Replication by elevation | Climate Zones | Specialization | Connectance | Nestedness | Modularity | Species Richness |
| --- | --- | --- | --- | --- | --- | --- | --- | --- | --- | --- | --- | --- | --- |
| Adedoja et al. 2018 | Jonaskop Mountain, Western Cape Province, South Africa | Cape Floristic Region Biodiversity Hotspot | 33˚58’10.67’’S | 19˚30’21.96’’E | Insecta: Hymenoptera (Bees and Wasps), Coleoptera, Diptera | 550-1576 m | Yes | Subtropic | No change | Decreases, increase at highest | No change | NA | mid-elev peak |
| Aguirre and Junker 2024 | National Park Hohe Tauern, Austria | Montane, subalpine, alpine forest | 47.0004° N | 12.5408° E | Insecta | 1179-2597 m | Yes | Temperate | Oscillating | NA | Increase | Oscillating | Decrease |
| Chesshire et al. 2024 | San Francisco Peaks, Arizona, USA | Ponderosa Pine, Mixed Conifer, Spruce Pine | 35.3408° N | 111.6832° W | Insecta: Hymenoptera (Bees, Wasps), Lepidoptera (Butterflies, Moths) | 2200-3100 m | No | Temperate | Decrease | Decrease | Increase | Decrease | mid-elev peak |
| Classen et al. 2020 | Mount Kilimanjaro, Tanzania | Colline savanna, maize fields, lower montane forests, agroforests, grassland, subalpine | 3.0674° S | 37.3556° E | Insecta: Hymenoptera (Bees, Wasps), Diptera (Syrphidae) | 993-4930 m | Yes | Paleotropic | Decrease | NA | Increase | NA | NA |
| Cristóbal-Perez et al. 2024 | Cerro de la Muerte, Talamanca Mountain Range, Costa Rica | Montane forest and Paramo | 09˚ 33’ N | 83˚ 44’ W | Insecta: Hymenoptera (Bees), Diptera, Coleoptera, Lepidoptera, Hemiptera | 3000-3500 m | No | Neotropic | Decrease | No change | Increase | Decrease | Decrease |
| Dzekashu et al. 2023 | Taita Hills (TH) and Murang'a (M), Kenya | Eastern Afromontane Biodiversity Hotspot | TH: −3°15′ to −4°0´S, M: 0°34′ to 1°5´S' | TH: 38°10′ to 39°03′°E, M: 36°43′ to 37°27′ E | Insecta: Hymenoptera (Bees) | 515-2600 m | No | Paleotropic | No change | Seasonal; decreasing dry season/increasing wet season | Increase | Seasonal; decreasing in wet season | decrease and mid-elev peak seasonally |
| Hoiss et al. 2015 | National Park Berchtesgaden, Germany | Grassland | 47.10°N | 12.15°E | Insecta: Diptera, Hymenoptera (Bees) | 600-2000 m | No | Temperate | Decrease | NA | NA | NA | NA |
| Lara-Romero et al. 2019 | Tenerife, Canary Islands, Spain | Oceanic high-mountain strato-volcano | 28°16ʹ15″N | 16°38ʹ21″W | Insecta: Coleoptera, Diptera, Hemiptera, Hymenoptera, Lepidoptera, and Orthoptera | 2350-3520 m | Yes | Subtropic | Decrease | Increase | NA | Decrease | decrease |
| Moreira et al. 2020 | South America Meta-Analysis |  | 0.04°S to 41.00°S | - | Aves: Apodiformes, Insecta: Coleoptera, Diptera, Hemiptera, Hymenoptera | 25 to 1020 m for Neotropics/Insecta portion of meta-analysis | Yes | Partially includes Neotropic | NA | Decrease | NA | NA | decrease |
| Pelayo et al. 2021 | Sierra de La Culata National Park, Venezuela | High Andean páramo | 8.7996° N | 70.9667° W | Aves: Apodiformes, Passeriformes; Insecta: Diptera, Hymenoptera (Bees, Wasps), Lepidoptera | 4207-4604m | Yes | Neotropic | Decrease; Increase at highest summit | Increase | Increase, Decrease at Highest | NA | decrease |
| Ramos-Jiliberto et al. 2010 | Lagunillas and Cordón del Cepo, Chile | Andean zone | 33˚ 36’ S | 70⁰ 17' W | Insecta: Hymenoptera (Bees), Diptera, Coleoptera, Lepidoptera (Butterflies); Aves: Apodiformes | 1800-3600m | Yes | Subtropic | NA | **Increase** | Decrease | **Decrease** |  |
| Wang et al. 2024 | Global Meta-Analysis | Global | Various | Various | Insecta: Hymenoptera, Diptera | Various | No | Global; mostly temperate | Increase | Decrease | Increase | Increase |  |

Table S2. Definitions of network metrics mentioned in manuscript.

| CONNECTANCE: | Connectance describes the proportion of realized interactions within the network, or the number of observed interactions, over the total number of possible interactions (Elle et al. 2012, Olesen et al. 2007), calculated as C=L/(A x P), where L is the observed amount of total links in the network and A and P are the total number of animal and plant species, respectively (Ramos-Jiliberto et al. 2010). The value of connectance is represented on a range from 0 to 1, where a score of 0 indicates no connectance in the network and 1 represents a completely connected network, where all possible interactions are detected and depicted. When the connectance score of a pollination network increases, the core number of links between generalist species increases. Conversely, connectance is expected to decrease when the network size is large and is dominated by select groups of species with highly specialized links (Kaiser-Bunbury and Blüthgen 2015). High network connectance suggests an overall generalist structure within the network and implies a higher degree of stability (Thébault and Fontaine 2010). However, the converse has also been shown, specifically in networks with higher connectance showing decreased stability in plant persistence. This work finds that connectance is often very low in natural plant-pollinator networks due to niche partitioning from adaptive foraging (Valdovinos et al. 2016). |
| --- | --- |
| H2’: | The index of specialization of the entire network (H2’) is defined as the degree of dependency on a few mutualistic partners (Kaiser-Bunbury and Blüthgen 2015). Specialization is the opposite of generalization, promoting interactions within a few exclusive partners. Specialization may also be calculated within species of a network (d’) differing from (H2’) as the latter compares specialization across multiple different networks (Blüthgen et al. 2006). The index of specialization ranges from 0 (complete generalization) to 1 (complete specialization). |
| MODULARITY: | Modularity describes the extent to which network structure is organized into modules (Olesen et al. 2007), where a highly modular network consists of many interactions with a few key species. Modularity is often used for detecting coevolutionary traits in pollination networks (Carstensen et al. 2016, Olesen et al. 2007) because core modules are associated with a similar pollination syndrome (Danieli-Silva et al. 2012). However, modularity is sensitive to network size as all networks with > 150 species are significantly modular. Conversely, networks < 50 species are never modular (Chesshire et al. 2021, Olesen et al. 2007), suggesting that an increase in the number of species significantly increases the chance of specialized or singleton interactions. Therefore, an increase in modularity is likely to imply an increase in specialization for the network structure. From a practical application, networks with high modularity are expected to be more robust to disturbance events (Olesen et al. 2007) as topological roles may dictate whether the network becomes fragmented, or a newly introduced species may coagulate the network structure. Modularity is represented on a range from 0 (no modular structures) to 1 (completely modular). |
| INTERACTION DIVERSITY: | The network property of interaction diversity is associated with the number of links in the network and the evenness of the interactions (Kaiser-Bunbury and Blüthgen 2015). Networks with a high interaction diversity imply higher community stability. Interaction evenness describes the homogeneity of all interaction frequencies. For example, a network with high interaction evenness will have a uniform spread of interactions, suggesting a generalist structure. In their review, Kaiser-Bunbury and Blüthgen (2015) described interaction evenness as an analog for connectance. |
| NESTEDNESS: | Nestedness describes a gradient where specialist interactions are subsets of generalist interactions (Cristóbal-Perez et al. 2024, Nielsen and Bascompte 2007, Tylianakis et al. 2010). In a highly nested network, specialists tend to share partners also interacting with generalists (Thébault and Fontaine 2010). One-to-one specialist relationships are likely to be highly impacted by disturbance and extinction events, but having a nested structure ensures their reciprocal partners are still able to interact with generalists. Tylianakis et al. (2010) describes nestedness in mutualistic networks as a buffer against extinctions and fluctuations. Nestedness scores range from 0 (not nested) to 100 (perfectly nested). This study focuses on the Weighted Nestedness metric based on Overlap and Decreasing Fill (WNODF), which takes into account the interaction frequencies of species in the network (Almeida-Neto and Ulrich 2011). |

Table S3. Plant species recorded along elevational gradient. Plant species highlighted in gray were involved in more than 3% of bee-plant interactions observed along the elevational gradient.

| Plant species | L | LM | M | MH | H | Grand Total |
| --- | --- | --- | --- | --- | --- | --- |
| *Abutilon pictum* | 0 | 0 | 0 | 2 | 5 | 7 |
| *Acnistus arborescens* | 0 | 5 | 0 | 0 | 32 | 37 |
| *Agapanthus praecox* | 0 | 0 | 0 | 0 | 3 | 3 |
| *Allamanda blanchetii* | 5 | 0 | 0 | 0 | 0 | 5 |
| *Allamanda cathartica* | 3 | 0 | 13 | 0 | 0 | 16 |
| *Alpinia spp.* | 0 | 1 | 0 | 0 | 0 | 1 |
| *Alstroemeria aurea* | 0 | 0 | 0 | 0 | 1 | 1 |
| *Amaranthus spinosus* | 0 | 3 | 0 | 0 | 0 | 3 |
| *Amaranthus spp.* | 0 | 1 | 0 | 0 | 40 | 41 |
| *Angelonia angustifolia* | 0 | 60 | 3 | 0 | 0 | 63 |
| *Anthurium andraeanum* | 0 | 0 | 1 | 0 | 0 | 1 |
| *Antigonon leptopus* | 49 | 0 | 0 | 0 | 0 | 49 |
| *Arachis glabrata* | 22 | 0 | 0 | 0 | 0 | 22 |
| *Arachis hypogaea* | 0 | 0 | 0 | 0 | 20 | 20 |
| *Argemone mexicana* | 0 | 0 | 0 | 0 | 1 | 1 |
| *Asclepias curassavica* | 0 | 0 | 1 | 0 | 18 | 19 |
| *Asystasia gangetica* | 4 | 4 | 29 | 40 | 0 | 77 |
| *Asystasia intrusa* | 0 | 0 | 3 | 0 | 0 | 3 |
| *Averrhoa carambola* | 19 | 18 | 0 | 0 | 0 | 37 |
| *Baltimora recta* | 0 | 34 | 0 | 0 | 0 | 34 |
| *Baltimora spp.* | 1 | 0 | 0 | 0 | 0 | 1 |
| *Bauhinia monandra* | 0 | 12 | 4 | 0 | 0 | 16 |
| *Bauhinia variegata* | 5 | 0 | 0 | 0 | 0 | 5 |
| *Begonia spp.* | 1 | 0 | 0 | 0 | 0 | 1 |
| *Bidens alba* | 0 | 0 | 0 | 0 | 1 | 1 |
| *Bidens pilosa* | 0 | 0 | 2 | 16 | 6 | 24 |
| *Bougainvillea spp.* | 2 | 7 | 7 | 7 | 9 | 32 |
| *Brassica rapa* | 0 | 0 | 0 | 11 | 0 | 11 |
| *Brassica spp.* | 0 | 0 | 20 | 0 | 0 | 20 |
| *Brugmansia suaveolens* | 0 | 0 | 0 | 0 | 11 | 11 |
| *Brunfelsia grandiflora* | 0 | 0 | 0 | 0 | 1 | 1 |
| *Buddleja davidii* | 0 | 0 | 1 | 0 | 9 | 10 |
| *Bunchosia grandulifera* | 0 | 29 | 0 | 0 | 0 | 29 |
| *Byrsonima crassifolia* | 0 | 34 | 0 | 0 | 0 | 34 |
| *Caesalpinia pulcherrima* | 48 | 206 | 18 | 0 | 0 | 272 |
| *Cajanus cajan* | 0 | 0 | 0 | 0 | 4 | 4 |
| *Calathea crotalifera* | 0 | 0 | 0 | 0 | 29 | 29 |
| *Calliandra haematocephala* | 0 | 0 | 4 | 0 | 0 | 4 |
| *Calliandra harrisii* | 0 | 0 | 8 | 0 | 0 | 8 |
| *Calliandra houstoniana* | 0 | 0 | 0 | 0 | 2 | 2 |
| *Calliandra spp.* | 0 | 0 | 4 | 0 | 0 | 4 |
| *Calliandra surinamensis* | 0 | 0 | 0 | 0 | 1 | 1 |
| *Canna indica* | 0 | 1 | 0 | 0 | 0 | 1 |
| *Canna spp.* | 0 | 0 | 0 | 0 | 2 | 2 |
| *Canna x generalis* | 0 | 0 | 1 | 0 | 0 | 1 |
| *Caperonia palustris* | 6 | 0 | 0 | 0 | 0 | 6 |
| *Capsicum annum* | 1 | 7 | 13 | 0 | 0 | 21 |
| *Catharanthus roseus* | 0 | 0 | 2 | 7 | 0 | 9 |
| *Celosia argentea* | 0 | 0 | 1 | 0 | 0 | 1 |
| *Centrosema molle* | 1 | 0 | 0 | 0 | 0 | 1 |
| *Cereus spp.* | 7 | 0 | 3 | 0 | 0 | 10 |
| *Chaenomeles cathayensis* | 0 | 0 | 4 | 0 | 0 | 4 |
| *Citrus spp.* | 0 | 53 | 1 | 12 | 2 | 68 |
| *Clitoria ternatea* | 1 | 0 | 0 | 0 | 0 | 1 |
| *Cnidoscolus spp.* | 0 | 0 | 0 | 0 | 3 | 3 |
| *Cocos nucifera* | 56 | 31 | 24 | 0 | 0 | 111 |
| *Codiaeum variegatum* | 2 | 0 | 0 | 0 | 0 | 2 |
| *Coffea arabica* | 0 | 0 | 0 | 30 | 0 | 30 |
| *Coix lacrima-jobi* | 0 | 0 | 0 | 0 | 4 | 4 |
| *Coleus scutellarioides* | 0 | 0 | 1 | 0 | 0 | 1 |
| *Commelina diffusa* | 0 | 0 | 1 | 0 | 0 | 1 |
| *Cordyline fruticosa* | 0 | 0 | 0 | 0 | 10 | 10 |
| *Coriandrum sativum* | 0 | 0 | 0 | 18 | 0 | 18 |
| *Cosmos bipinnatus* | 0 | 0 | 0 | 1 | 0 | 1 |
| *Cosmos sulphureus* | 0 | 157 | 26 | 23 | 25 | 231 |
| *Crocosmia x crocosimiiflora* | 0 | 0 | 0 | 0 | 1 | 1 |
| *Crotalaria juncea* | 0 | 0 | 0 | 1 | 0 | 1 |
| *Croton hirtus* | 0 | 22 | 0 | 0 | 0 | 22 |
| *Cryptostegia grandiflora* | 4 | 0 | 2 | 0 | 0 | 6 |
| *Cucurbita moschata* | 11 | 0 | 0 | 0 | 0 | 11 |
| *Cuphea hyssopifolia* | 11 | 0 | 34 | 0 | 0 | 45 |
| *Cynoglossum amabile* | 0 | 0 | 0 | 7 | 0 | 7 |
| *Cyperus entrerianus* | 15 | 0 | 0 | 0 | 0 | 15 |
| *Dahlia pinnata* | 0 | 0 | 2 | 0 | 0 | 2 |
| *Duranta erecta* | 11 | 1 | 8 | 27 | 52 | 99 |
| *Eleutheranthera ruderalis* | 0 | 5 | 0 | 0 | 0 | 5 |
| *Emilia fosbergii* | 0 | 2 | 0 | 0 | 0 | 2 |
| *Erigeron annus* | 0 | 0 | 0 | 0 | 5 | 5 |
| *Erigeron sumatrensis* | 0 | 4 | 0 | 0 | 0 | 4 |
| *Eryngium foetidum* | 3 | 23 | 8 | 0 | 0 | 34 |
| *Euphorbia geroldii* | 11 | 17 | 26 | 13 | 2 | 69 |
| *Euphorbia graminea* | 0 | 0 | 7 | 4 | 16 | 27 |
| *Euphorbia milii* | 5 | 6 | 15 | 0 | 0 | 26 |
| *Euphorbia nutans* | 15 | 0 | 0 | 0 | 0 | 15 |
| *Euphorbia spp.* | 0 | 0 | 0 | 11 | 0 | 11 |
| *Fuchsia boliviana* | 0 | 0 | 0 | 0 | 4 | 4 |
| *Fuchsia triphylla* | 0 | 0 | 0 | 0 | 1 | 1 |
| *Galinsoga quadriradiata* | 0 | 0 | 0 | 2 | 0 | 2 |
| *Globba spp.* | 0 | 0 | 1 | 0 | 0 | 1 |
| *Gomphrena globosa* | 0 | 0 | 6 | 0 | 0 | 6 |
| *Gongora armeniaca* | 0 | 0 | 1 | 0 | 0 | 1 |
| *Grevillea banksii* | 0 | 2 | 0 | 0 | 0 | 2 |
| *Hamelia patens* | 0 | 0 | 17 | 34 | 123 | 174 |
| *Hampea appendiculata* | 0 | 0 | 0 | 0 | 5 | 5 |
| *Heliconia spp.* | 40 | 1 | 0 | 2 | 5 | 48 |
| *Heliotropium indicum* | 0 | 9 | 0 | 0 | 0 | 9 |
| *Hemerocallis spp.* | 0 | 0 | 0 | 0 | 1 | 1 |
| *Hibiscus rosa-sinensis* | 0 | 1 | 0 | 0 | 0 | 1 |
| *Hibiscus schizopetalus* | 0 | 4 | 0 | 0 | 0 | 4 |
| *Hibiscus spp.* | 31 | 21 | 0 | 3 | 2 | 57 |
| *Hippeastrum puniceum* | 0 | 0 | 3 | 0 | 0 | 3 |
| *Hippeastrum spp.* | 0 | 0 | 0 | 0 | 2 | 2 |
| *Hydrangea spp.* | 0 | 4 | 19 | 10 | 6 | 39 |
| *Hylocereus undatus* | 0 | 0 | 8 | 0 | 0 | 8 |
| *Impatiens spp.* | 0 | 7 | 8 | 67 | 6 | 88 |
| *Impatiens walleriana* | 0 | 0 | 0 | 3 | 0 | 3 |
| *Iochroma arborescens* | 0 | 0 | 0 | 0 | 19 | 19 |
| *Ipomea aurantiaca* | 0 | 0 | 0 | 0 | 2 | 2 |
| *Ixora spp.* | 103 | 17 | 14 | 0 | 0 | 134 |
| *Jatropha gossypifolia* | 0 | 40 | 0 | 0 | 0 | 40 |
| *Jatropha integerrima* | 10 | 15 | 50 | 0 | 0 | 75 |
| *Jatropha multifada* | 5 | 0 | 172 | 9 | 0 | 186 |
| *Jatropha podagrica* | 5 | 0 | 0 | 0 | 0 | 5 |
| *Lagerstroemia indica* | 88 | 1 | 14 | 0 | 0 | 103 |
| *Lagerstroemia speciosa* | 18 | 0 | 0 | 0 | 0 | 18 |
| *Lantana camara* | 0 | 11 | 5 | 18 | 8 | 42 |
| *Lantana spp.* | 0 | 0 | 0 | 1 | 1 | 2 |
| *Lantana velutina* | 2 | 0 | 0 | 0 | 0 | 2 |
| *Lasianthaea fruticosa* | 0 | 0 | 3 | 1 | 0 | 4 |
| *Leucanthemum maximum* | 0 | 0 | 0 | 0 | 1 | 1 |
| *Leuenbergeria bleo* | 2 | 0 | 0 | 0 | 0 | 2 |
| *Lippia alba* | 10 | 0 | 86 | 10 | 12 | 118 |
| *Lycianthes ratonnetii* | 0 | 1 | 0 | 0 | 0 | 1 |
| *Malpighia glabra* | 0 | 0 | 13 | 0 | 0 | 13 |
| *Malvaviscus spp.* | 0 | 0 | 0 | 3 | 0 | 3 |
| *Melampodium perfoliatum* | 0 | 25 | 0 | 3 | 0 | 28 |
| *Melampodium spp.* | 2 | 0 | 0 | 0 | 0 | 2 |
| *Miconia xalapensis* | 0 | 0 | 0 | 2 | 0 | 2 |
| *Mimosa pudica* | 0 | 0 | 0 | 0 | 2 | 2 |
| *Mirabilis jalapa* | 0 | 2 | 3 | 0 | 0 | 5 |
| *Mortoniodendron guatamalense* | 0 | 0 | 0 | 10 | 0 | 10 |
| *Muntingia calabura* | 0 | 13 | 0 | 0 | 0 | 13 |
| *Murraya paniculata* | 6 | 0 | 0 | 0 | 0 | 6 |
| *Musa spp.* | 154 | 65 | 46 | 71 | 86 | 422 |
| *Ocimum basilicum* | 4 | 10 | 42 | 26 | 19 | 101 |
| *Pachystachys lutea* | 10 | 0 | 4 | 3 | 0 | 17 |
| *Palicourea padifolia* | 0 | 0 | 0 | 0 | 9 | 9 |
| *Palm spp.* | 0 | 0 | 27 | 0 | 0 | 27 |
| *Pentas lanceolata* | 0 | 0 | 0 | 29 | 29 | 58 |
| *Phaseolus spp.* | 0 | 0 | 0 | 1 | 0 | 1 |
| *Phaseolus vulgaris* | 0 | 0 | 0 | 4 | 0 | 4 |
| *Plectranthus scutellariodes* | 0 | 1 | 0 | 0 | 0 | 1 |
| *Pleroma heteromallum* | 0 | 0 | 1 | 4 | 0 | 5 |
| *Pleroma urvilleanum* | 0 | 11 | 13 | 0 | 0 | 24 |
| *Portulaca spp.* | 84 | 100 | 58 | 17 | 0 | 259 |
| *Psidium cattleyanum* | 0 | 0 | 0 | 0 | 8 | 8 |
| *Psidium guajava* | 0 | 0 | 0 | 0 | 1 | 1 |
| *Psychotria spp.* | 0 | 0 | 0 | 3 | 0 | 3 |
| *Raphanus sativus* | 0 | 0 | 0 | 16 | 0 | 16 |
| *Rhododendron indicum* | 5 | 0 | 0 | 0 | 1 | 6 |
| *Rhyncospora colorata* | 0 | 14 | 0 | 0 | 0 | 14 |
| *Rhyncospora spp.* | 29 | 5 | 0 | 0 | 0 | 34 |
| *Rosa chinensis* | 0 | 0 | 8 | 0 | 1 | 9 |
| *Rosa setigera* | 0 | 0 | 0 | 0 | 1 | 1 |
| *Rosa spp.* | 0 | 0 | 5 | 0 | 0 | 5 |
| *Rotheca myricoides* | 0 | 6 | 0 | 0 | 0 | 6 |
| *Rubus spp.* | 0 | 0 | 0 | 1 | 0 | 1 |
| *Ruellia simplex* | 0 | 1 | 12 | 0 | 0 | 13 |
| *Salvia coccinea* | 0 | 0 | 3 | 1 | 0 | 4 |
| *Salvia elegans* | 0 | 0 | 0 | 0 | 37 | 37 |
| *Salvia splendens* | 0 | 0 | 10 | 0 | 2 | 12 |
| *Salvia spp.* | 0 | 0 | 0 | 4 | 0 | 4 |
| *Sambucus spp.* | 0 | 0 | 0 | 0 | 20 | 20 |
| *Satureja viminea* | 0 | 0 | 0 | 5 | 0 | 5 |
| *Scoparia dulcis* | 1 | 0 | 0 | 0 | 0 | 1 |
| *Sechium edule* | 0 | 0 | 1 | 131 | 3 | 135 |
| *Senna siamea* | 0 | 7 | 0 | 0 | 0 | 7 |
| *Sida spp.* | 5 | 0 | 0 | 0 | 0 | 5 |
| *Solanum americana* | 0 | 0 | 3 | 1 | 0 | 4 |
| *Solanum lycopersicum* | 0 | 4 | 5 | 18 | 1 | 28 |
| *Solanum umbellatum* | 0 | 0 | 0 | 0 | 2 | 2 |
| *Solanum wendlandii* | 0 | 0 | 12 | 0 | 0 | 12 |
| *Spathiphyllum wallisi* | 0 | 0 | 0 | 2 | 0 | 2 |
| *Sphagneticola trilobata* | 0 | 0 | 0 | 3 | 5 | 8 |
| *Stachytarpheta spp.* | 0 | 30 | 20 | 47 | 110 | 207 |
| *Streptosolen jamesonii* | 0 | 0 | 0 | 17 | 10 | 27 |
| *Strobilanthes hamiltoniana* | 0 | 0 | 3 | 0 | 0 | 3 |
| *Syzygium malaccense* | 0 | 0 | 0 | 3 | 0 | 3 |
| *Tagetes erecta* | 1 | 0 | 0 | 0 | 0 | 1 |
| *Tagetes lucida* | 0 | 0 | 0 | 0 | 1 | 1 |
| *Talinum paniculatum* | 0 | 0 | 1 | 0 | 0 | 1 |
| *Terminalia catappa* | 0 | 35 | 0 | 0 | 0 | 35 |
| *Terminalia spp.* | 0 | 17 | 0 | 0 | 0 | 17 |
| *Thunbergia alata* | 0 | 0 | 0 | 4 | 2 | 6 |
| *Thunbergia erecta* | 0 | 0 | 3 | 19 | 0 | 22 |
| *Thunbergia mysorensis* | 0 | 0 | 0 | 0 | 29 | 29 |
| *Tithonia rotundifolia* | 0 | 0 | 2 | 0 | 0 | 2 |
| *Torenia fourneri* | 0 | 0 | 4 | 0 | 0 | 4 |
| *Tridax procumbens* | 5 | 0 | 0 | 0 | 0 | 5 |
| *Trimezia steyermarkii* | 6 | 9 | 1 | 18 | 1 | 35 |
| *Tripogandra serrulata* | 0 | 0 | 0 | 11 | 0 | 11 |
| *Turnera subulata* | 188 | 13 | 0 | 11 | 0 | 212 |
| *Turnera ulmifolia* | 12 | 0 | 6 | 21 | 0 | 39 |
| *Verbena litoralis* | 0 | 0 | 1 | 0 | 0 | 1 |
| *Verbena spp.* | 0 | 0 | 0 | 0 | 3 | 3 |
| *Youngia japonica* | 0 | 0 | 0 | 0 | 5 | 5 |
| *Zantedescia aethopica* | 0 | 0 | 1 | 0 | 0 | 1 |
| *Zanthoxylum fagara* | 0 | 0 | 0 | 82 | 0 | 82 |
| *Zea mays* | 0 | 20 | 0 | 12 | 0 | 32 |
| *Zephyranthes carinata* | 0 | 0 | 0 | 0 | 1 | 1 |
| *Zephyranthes spp.* | 0 | 0 | 0 | 1 | 0 | 1 |
| *Zinnia elegans* | 6 | 1 | 47 | 1 | 7 | 62 |
| *Zinnia peruviana* | 0 | 0 | 4 | 0 | 0 | 4 |
| *Zinnia spp.* | 2 | 0 | 4 | 0 | 0 | 6 |
| Grand Total | 1153 | 1263 | 1067 | 850 | 909 | 5242 |

Table S4. Bee-plant Network metrics full data including null model means and s.d. for each metric at each plot.

| site | Elevation | NH2 | NQ | NC | NN | DH2 | DQ | DC | DN | sdH2 | sdQ | sdC | sdN |
| --- | --- | --- | --- | --- | --- | --- | --- | --- | --- | --- | --- | --- | --- |
| HotelBelmar | H | 0.114 | 0.128 | 0.1344 | 32.851 | 0.1457 | 0.1317 | -0.0376 | -19.671 | 0.036 | 0.0124 | 0.004 | 3.63 |
| HotelElBosque | H | 0.185 | 0.3032 | 0.1428 | 19.101 | 0.3177 | 0.1995 | -0.0348 | -6.311 | 0.028 | 0.0197 | 0.009 | 2.517 |
| Mariposa | H | 0.165 | 0.236 | 0.1699 | 28.767 | 0.291 | 0.22 | -0.0545 | -22.832 | 0.036 | 0.0163 | 0.008 | 4.142 |
| Mayra | H | 0.165 | 0.2634 | 0.13669 | 23.376 | 0.3544 | 0.256 | -0.05389 | -12.108 | 0.019 | 0.0175 | 0.006 | 2.574 |
| Peter | H | 0.134 | 0.2066 | 0.1625 | 29.898 | 0.27 | 0.1974 | -0.05254 | -13.77 | 0.032 | 0.0203 | 0.008 | 4.651 |
| Tercicio | H | 0.174 | 0.2996 | 0.14408 | 20.167 | 0.3 | 0.1744 | -0.04788 | -9.444 | 0.026 | 0.0191 | 0.009 | 3.201 |
| Guillermo | L | 0.131 | 0.204 | 0.1375 | 28.658 | 0.5449 | 0.4719 | -0.0892 | -22.249 | 0.016 | 0.0139 | 0.0054 | 3.036 |
| LilaCruz | L | 0.124 | 0.1942 | 0.16096 | 34.369 | 0.4629 | 0.3927 | -0.09156 | -21.531 | 0.011 | 0.012 | 0.004 | 2.8415 |
| LupesMom | L | 0.094 | 0.1434 | 0.14815 | 43.215 | 0.4225 | 0.3731 | -0.08015 | -30.209 | 0.013 | 0.0122 | 0.004 | 3.465 |
| MariaRamirez | L | 0.134 | 0.2283 | 0.1629 | 27.98 | 0.4705 | 0.3762 | -0.082 | -17.71 | 0.0202 | 0.0159 | 0.007 | 3.646 |
| Dimas | LM | 0.123 | 0.2076 | 0.18135 | 32.476 | 0.4568 | 0.3722 | -0.08905 | -22.826 | 0.023 | 0.0176 | 0.009 | 4.553 |
| DonAristetes | LM | 0.108 | 0.1457 | 0.09251 | 31.033 | 0.3164 | 0.2787 | -0.04571 | -22.1014 | 0.012 | 0.0097 | 0.002 | 2.496 |
| Josefina | LM | 0.156 | 0.2421 | 0.17697 | 29.427 | 0.3685 | 0.2824 | -0.09097 | -17.73 | 0.017 | 0.0153 | 0.006 | 2.621 |
| Lucy | LM | 0.163 | 0.2819 | 0.17669 | 23.848 | 0.4351 | 0.3162 | -0.07079 | -16.423 | 0.035 | 0.0237 | 0.0109 | 3.629 |
| Oscar | LM | 0.149 | 0.2491 | 0.18116 | 25.989 | 0.5309 | 0.4308 | -0.11083 | -17.0093 | 0.018 | 0.0169 | 0.007 | 2.782 |
| Pipe | LM | 0.115 | 0.168 | 0.1865 | 41.753 | 0.413 | 0.36 | -0.0712 | -20.605 | 0.034 | 0.0242 | 0.009 | 6.429 |
| Blanca | M | 0.147 | 0.2339 | 0.1369 | 25.246 | 0.4608 | 0.3739 | -0.0786 | -18.318 | 0.021 | 0.0155 | 0.006 | 2.992 |
| Memei | M | 0.168 | 0.3408 | 0.1286 | 14.876 | 0.4254 | 0.2526 | -0.037 | -1.422 | 0.024 | 0.0194 | 0.007 | 2.282 |
| Ramon | M | 0.155 | 0.2333 | 0.19485 | 35.655 | 0.511 | 0.4327 | -0.11415 | -26.625 | 0.019 | 0.0167 | 0.0065 | 3.245 |
| Victor | M | 0.194 | 0.2935 | 0.15819 | 21.59 | 0.3425 | 0.243 | -0.06415 | -12.85 | 0.023 | 0.0183 | 0.008 | 2.713 |
| Yolanda | M | 0.175 | 0.239 | 0.15211 | 27.537 | 0.3273 | 0.2633 | -0.07331 | -17.499 | 0.0255 | 0.0175 | 0.0075 | 3.279 |
| Zulay | M | 0.167 | 0.2896 | 0.1357 | 18.859 | 0.402 | 0.2794 | -0.0486 | -7.343 | 0.0199 | 0.0162 | 0.007 | 2.578 |
| Carlos | MH | 0.097 | 0.1712 | 0.2125 | 43.969 | 0.428 | 0.3538 | -0.0865 | -29.549 | 0.0204 | 0.0203 | 0.007 | 4.641 |
| Danilo | MH | 0.198 | 0.2574 | 0.1689 | 28.529 | 0.4073 | 0.3479 | -0.089 | -19.672 | 0.039 | 0.0246 | 0.0102 | 4.897 |
| Elisa | MH | 0.183 | 0.3555 | 0.15743 | 19.531 | 0.4023 | 0.2298 | -0.05343 | -6.087 | 0.038 | 0.0283 | 0.011 | 3.376 |
| Maco | MH | 0.159 | 0.2091 | 0.13378 | 28.727 | 0.1836 | 0.1335 | -0.03768 | -14.998 | 0.032 | 0.0147 | 0.006 | 3.722 |
| Oldemar | MH | 0.1596 | 0.1914 | 0.157 | 30.875 | 0.24836 | 0.21656 | -0.0528 | -15.124 | 0.023 | 0.0139 | 0.005 | 3.752 |
| Sergio | MH | 0.168 | 0.2777 | 0.14002 | 21.868 | 0.3259 | 0.2162 | -0.01852 | -6.608 | 0.028 | 0.0199 | 0.008 | 2.761 |

Figure S1. Distance-based redundancy analysis ordispider grouped by home garden. Each black circle represents one of the home gardens. CAP1 represents elevation and increases as represented by mid-high (1100-1200 m) and high-elevation sites (1400-1500 m).

Figure S2. Mean ± s.e. of plant species richness in bipartite networks by elevational band.
